# The Versatility of Antioxidant Assays in Food Science and Safety—Chemistry, Applications, Strengths, and Limitations

**DOI:** 10.3390/antiox9080709

**Published:** 2020-08-05

**Authors:** Nabeelah Bibi Sadeer, Domenico Montesano, Stefania Albrizio, Gokhan Zengin, Mohamad Fawzi Mahomoodally

**Affiliations:** 1 Department of Health Sciences; Faculty of Science, University of Mauritius, Réduit 80837, Mauritius; nabeelah.sadeer1@umail.uom.ac.mu; 2 Department of Pharmaceutical Sciences, Section of Food Science and Nutrition, University of Perugia, via S. Costanzo, 06126 Perugia, Italy; domenico.montesano@unipg.it; 3 Department of Pharmacy, University of Naples “Federico II”, via D. Montesano 49, 80131 Naples, Italy; 4 Consorzio Interuniversitario INBB—Viale Medaglie d’Oro, 305, I-00136 Rome, Italy; 5 Department of Biology, Science Faculty, Selcuk University, 42250 Konya Campus, Turkey; gokhanzengin@selcuk.edu.tr

**Keywords:** antioxidants, free radicals, oxidative stress, spectrophotometer, limitations, chemical reactions, colorimetry

## Abstract

Currently, there is a growing interest in screening and quantifying antioxidants from biological samples in the quest for natural and effective antioxidants to combat free radical-related pathological complications. Antioxidant assays play a crucial role in high-throughput and cost-effective assessment of antioxidant capacities of natural products such as medicinal plants and food samples. However, several investigators have expressed concerns about the reliability of existing in vitro assays. Such concerns arise mainly from the poor correlation between in vitro and in vivo results. In addition, in vitro assays have the problem of reproducibility. To date, antioxidant capacities are measured using a panel of assays whereby each assay has its own advantages and limitations. This unparalleled review hotly disputes on in vitro antioxidant assays and elaborates on the chemistry behind each assay with the aim to point out respective principles/concepts. The following critical questions are also addressed: (1) What make antioxidant assays coloured? (2) What is the reason for working at a particular wavelength? (3) What are the advantages and limitations of each assay? and (4) Why is a particular colour observed in antioxidant–oxidant chemical reactions? Furthermore, this review details the chemical mechanism of reactions that occur in each assay together with a colour ribbon to illustrate changes in colour. The review ends with a critical conclusion on existing assays and suggests constructive improvements on how to develop an adequate and universal antioxidant assay.

## 1. Introduction

Our life relies on a well-designed and orchestrated series of naturally occurring chemical reactions. The presence of billions of cells in our body is perpetually under threat of being harmed by radicals that can lead to the development of diseases. Diseases are not developed overnight. One of the main causes of diseases is related to ‘oxidative stress’ involving free radicals. Oxidative stress is the most inspected stress that disturbs the normal functioning of cells. It is responsible for scads of cell damage, leading to numerous degenerative diseases including neurodegenerative disorders (Alzheimer’s disease, Parkinson’s disease), cancers, cardiovascular diseases, retinopathy, and dermatological diseases. As a normal defence mechanism, our body reacts to any given stress to ascertain a healthy cellular homeostasis [1]. However, antioxidant enzymes present are sometimes not enough to combat free radicals. Thus, it is vital to either consume foods rich in antioxidants or alternatively, rely on medicines for the prevention and treatment of degenerative disorders. Despite free radicals causing a panoply of diseases, it is important to remember that they also show interesting therapeutic effects, especially in antimicrobial applications. For example, the free radical-releasing system is becoming an emerging strategy to combat antibiotic resistance and biofilm formations [2]. Free radical treatment is also recognized as an effective cancer treatment [3], although in some cases free radicals could be the leading cause of cancer. 

There has been an upsurge of interest in free radical chemistry since the past decades. At the time of writing, research studies conducted in various fields, regardless of whether the studies are food-related or plant-related, all samples are scrutinized for their antioxidant activities as a preliminary screening in the pursuit of novel compounds with powerful antioxidant properties [4,5]. Screening of biological samples for antioxidant capacities is done using a series of assays instead of relying on only one assay. This is because Opitz et al. [6], in their book chapter, have mentioned that one assay does not give realistic results compared to a series of assays involving different chemical reactions. It is acknowledged that published results are inconclusive and it is difficult to make comparisons between different research groups [7]. In addition, food and nutraceutical industries cannot perform strict quality control for antioxidant products [8]. 

The limitations and metabolism of antioxidants still represent a challenge for future research in the free radical chemistry field and thus, researchers are trying to search for alternatives or solutions to overcome such limitations. Some general limitations include: (i) In terms of neuroprotection, antioxidants do not deliver appropriate and effective protection solely due to the blood–brain barrier [9], (ii) dietary antioxidants are more sensitive in mice compared to humans. Thus, it is important to consider this fact before any clinical trials [10], (iii) Another limitation is linked with cell cultures. Sometimes during in vitro testing, antioxidants react with the reagents present in the reaction mixture, giving rise to erroneous results [7]. It is believed that the biggest problem lies in the lack of a validated and universal assay that can reliably measure the antioxidant capacities of foods and other biological samples. Interestingly, as well stressed in a review compiled by Granato et al. [11], it was mentioned that compounds measured in foods are not necessarily representative of those which are active in humans. For instance, after the consumption of blueberries, the presence of phenolic acids could be detected in the blood while noted absent in other consumers, since the compounds occurred as metabolites. Thus, it is understood that since complex interactions are involved among the intrinsic and extrinsic factors present in food and other biological matrices, antioxidant activity cannot be measured using simple chemical reactions in a test tube alone.

So far, there are piecemeal reviews on antioxidants elaborating on either one or a few assays. For instance, Re et al. [12] have reviewed an improved version of the ABTS radical cation decolorization assay. Huang, Ou, and Prior [8] have evaluated several antioxidant assays in terms of their kinetics of autoxidation. Alam et al. [13] have reviewed differences between in vivo and in vitro methods evaluating antioxidant activity. Carocho et al. [14] have compiled information on antioxidants in terms of their application in foods as preservatives. Ratnam et al. [15] have documented the role of antioxidants in a pharmaceutical perspective. Carocho and Ferreira [7] have published a review on antioxidants and pro-oxidants, including certain controversies. Kim et al. [16] have focused on the vitamin C equivalent antioxidant capacity (VCEAC) of phenolic phytochemicals, among others. After searching the existing literature, it is noticed that there is no review that systematically details the chemical reactions of each antioxidant assay. Additionally, no review has focused on the strengths and limitations of each assay or explained reasons behind the development of colours in such assays. Furthermore, consolidated improvements have not been suggested yet to develop a new and universal antioxidant assay. Therefore, such research gap has fuelled the need to present a review including all these missing aspects. The aim of the present review is not to be repetitive but attempts to provide a more informative, authoritative, and comparative coverage on the chemistry behind antioxidant assays, including a brief history on the development of different assays, explaining the principle, general concept of each assay, reasons why certain reagents are used, chemical reactions that occur are detailed, colour change developed in each assay, and the reason why absorbance is read at a particular wavelength with a spectrophotometer. The strengths and limitations of each assay are also listed, highlighting some key improvements to consider while validating a novel antioxidant method. 

## 2. Review Methodology

The relevant literature was collected by searching scientific electronic databases, namely ScienceDirect, Scopus, PubMed, Web of Science, and Google Scholar. Keywords such as antioxidants, free radicals, antioxidants assays/methods, antioxidant enzymes, chemical reactions, mechanism of reactions, wavelength, chemical reactions, colour change, chromogens, complexes, absorption, strengths, and limitations were used in the search process. Each antioxidant assay was described in terms of who has developed the assay, when the assay was developed, the principle behind the assay, chemical reactions, a detailed mechanism of reactions, colour change, strengths, and limitations. Chemical structures presented in the mechanism of reactions were drawn with ChemDraw Ultra 12.0.

## 3. Chemistry of Antioxidant Methods

Generally, antioxidant assays are conducted using appropriate traditional methodologies, collecting and processing the data in terms of % inhibition or the equivalent of standards, and finally, interpreting the results. The chemistry occurring in each assay tends to be ignored. We do not know why a certain type of assays is measured at a particular wavelength (λ), why radicals/probes are coloured, or why they change colour upon reactions. The following sections attempt to answer these questions, as the current review aims to provide the chemistry behind each antioxidant (AO) reaction.

### 3.1. Why Are Antioxidant Assays Coloured?

Colour is the product of electronic transitions in atoms or molecules and is an indicator of the physical properties of chemical substances at the atomic level. A change in the electronic transitions results in a change in the light absorbed by the molecules and subsequently, causes a change in colour. The coloured complex formed in AO assays is called a charge–transfer (CT) complex or electron–donor–acceptor complex. A CT complex is the association of two or more molecules, or different parts of one molecule, in which a fraction of electronic charge is transferred between the molecular entities (i.e., the radical and AO). This transfer results in an electrostatic force of attraction (J) between the radical and AO providing a stabilizing force for the CT complex. For instance, in the 2,2-diphenyl-1-picrylhydrazyl (DPPH) assay, the unpaired electron in DPPH^•^ exhibits an intense deep purple colour charge–transfer band at 517 nm, however, while pairing up with another electron, a change in colour is observed resulting in pale yellow. This permits us to answer the question why there is a colour change in AO assays. 

### 3.2. What Is the Reason for Working at a Particular Wavelength?

After receiving an electron, many complexes enter an excitation state. The excitation energy required for an electron to jump from one energy level to another often falls in the visible region of the electromagnetic spectrum, which consequently, results in the formation of intensely coloured complexes. The absorption bands are usually referred to as charge–transfer bands (CT bands). The absorption wavelength of the CT bands is distinctive in terms of the types of donor and acceptor involved. The electron donating power of the donor (E_I_) is referred to as its ionization energy, which is the energy needed to remove the most loosely bound electron from a neutral atom/or molecule. On the other hand, the electron accepting power of the acceptor (E_A_) is determined by its electron affinity, which is defined as the energy released when an electron is added to a neutral atom/or molecule to form an anion. The overall energy difference, denoted as ∆E, is the energy gained during the charge transfer:

∆E = E_A_ − E_I_ + J

where J is the electrostatic force of attraction. It is noteworthy to point out that this energy difference is directly related to a specific CT band in the electromagnetic spectrum which explains why it is important to work at a particular wavelength. 

### 3.3. Why Is a Particular Colour Observed in an Antioxidant–Oxidant Chemical Reaction?

**Figure 1:**
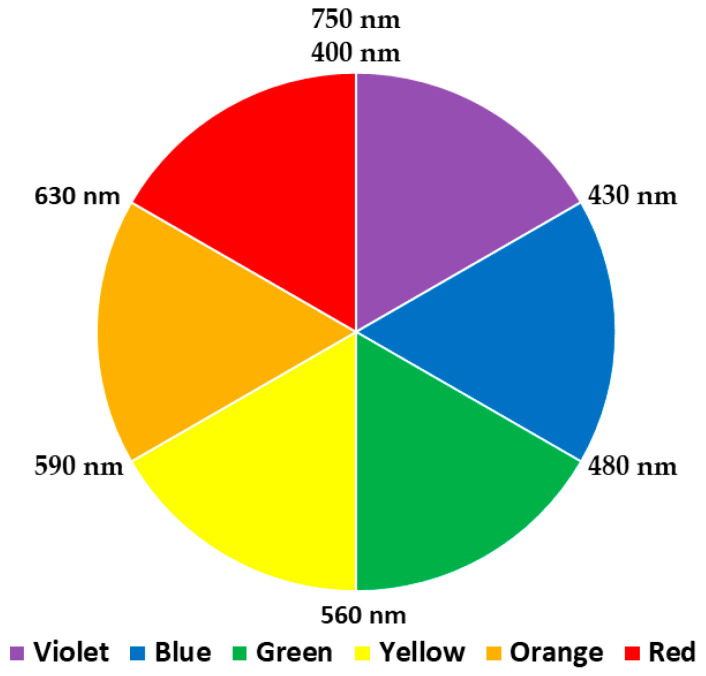
Colour wheel.

Light is a mixture of colours and the visible region in an electromagnetic spectrum is made up of different colours, namely red, orange, yellow, green, blue, and violet, covering a wavelength region of 400 to 750 nm, as represented by the colour wheel in Figure 1. When a molecule absorbs light at a particular wavelength, the colour that appear is the complementary colour on the colour wheel. For example, DPPH gives a deep purple appearance because it absorbs a photon of light at 515–517 nm, 2,2-azino-bis(3-ethylbenzothiazoline-6-sulfonic acid) (ABTS) absorbs at 734 nm to give a pale blue colour, and so on.

## 4. Mechanism of Action of Antioxidants

Antioxidant assays are based on a concept called total antioxidant capacity (TAC). TAC is measured as the amount of free radicals quenched by a test solution used to determine the AO capacity of a biological sample. Depending on the mechanism of chemical reactions involved, TAC assays can be further categorized as: (i) single electron transfer (SET), (ii) hydrogen atom transfer (HAT) reaction-based assays, or (iii) chelation of transition metals [17]. 

The single electron transfer (SET) mechanism involves a redox (reduction–oxidation) reaction with an oxidant (also known as the probe or radical) as an indicator of reaction endpoint. Hydrogen atom transfer (HAT) assays involve a synthetic radical generator, an oxidizable probe, and an antioxidant. Both SET and HAT reaction-based assays measure the radical scavenging capacity instead of the preventive capacity of a sample [8,18]. SET-based assays measure the antioxidant’s reducing capacity, while HAT-based assays quantify hydrogen atom donating capacity [19]. In SET assays, AO gives an electron to the radical to stabilize it. The transfer of an electron from AO to the radical will cause a change in colour of the radical. The intensity of the colour change is proportional to the concentration of AO present in the reaction mixture. The reaction end point is reached when no change in colour is observed [8], i.e., when electron transfer has stopped. 

In addition to SET and HAT mechanisms, the third type of mechanism of action of AOs is their ability to chelate transition metals, namely Zn^2+^, Fe^2+^, and Cu^2+^. The chelation of transition metals can also be considered to estimate the AO capacity of an extract or compound. Several lines of evidence extracted from the recent literature have demonstrated that transition metals such as Fe^2+^ and Cu^2+^ are responsible for the pathogenesis of numerous diseases, including neurodegenerative (Alzheimer’s, Parkinson’s) and cardiovascular diseases [17]. 

Various techniques have been developed to measure antioxidant capacities of biological samples, including plant extracts and food samples. The following sections will discuss these techniques as well as various AO assays in terms of the chemical reactions involved, mechanism of each reaction, colour change of probe, strengths and limitations of each assay. 

## 5. Different Techniques Used to Measure Antioxidant Activities

There are numerous analytical techniques available to measure the antioxidant property of samples. The different techniques fall into three main categories, namely spectrometry, electrochemical technique, and chromatography. Each one of them is discussed in Table 1. However, in this review, antioxidant assays using colorimetry as a measure of antioxidant properties are appraised, since they are the most accessible and commonly used methods to evaluate the antioxidant activities of biological samples. 

## 6. Folin–Ciocalteu Assay

The Folin–Ciocalteu (F–C) assay is the most commonly used assay to determine the total phenolic content in various plant or food samples. Phenolic compounds of chemo-preventive and therapeutic values are of scientific interest in the management of countless chronic diseases since 1990. The major contributors of antioxidant capacity of fruit, vegetable, grain, or plant samples are phenolic compounds. The F–C assay is a colorimetry method based on SET reactions between the F–C reagent and phenolic compounds [21]. Phenolic compounds are good oxygen radical scavengers, since the electron reduction potential of phenolic radical is lower than that of oxygen radicals and also, phenoxyl radicals are less reactive than oxygen radicals. Thus, scavenging reactive oxygen radicals by phenolic compounds ceased further oxidative reactions [22]. The F–C assay was developed to improve the Folin–Denis (F-D) assay, which was initially designed to determine total protein concentration by measuring tryptophan and tyrosine contents. Later, it was found that F–C was more sensitive and reproducible than the F–D assay [22]. However, the F–C assay is non-specific, since other substances, namely reducing sugars and ascorbic acid which are highly abundant in plant food extracts, can reduce F–C reagent, leading to biased F–C results [22]. 

**Figure 2:**
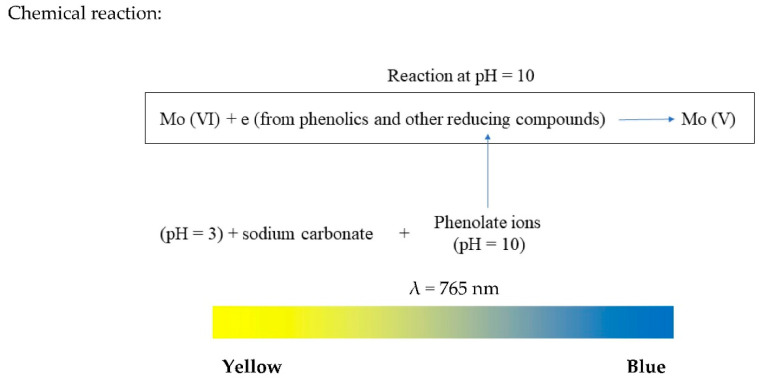
Folin–Ciocalteu (F–C) assay.

F–C reagent is prepared by dissolving 100 g of sodium tungstate (Na_2_WO_4_·2H_2_O) and 25 g sodium molybdate (Na_2_MoO_4_·2H_2_O) in 700 mL of distilled water. About 50 mL of concentrated HCl and 50 mL of 85% phosphoric acid are added to acidify the solution. The acidified solution is boiled for 10 h and allowed to cool before adding 150 g Li_2_SO_4_·4H_2_O. The resulting solution, which is the F–C reagent, develops an intense yellow colour [22]. The chemistry of F–C reagent is still unclear and is believed to be composed of heteropoly-phosphotungstates/molybdates [8]. During F–C assay, the reaction between F–C reagent and phenolic compounds occurs at alkaline medium (~pH 10), which is reached by adding sodium carbonate (Na_2_CO_3_). Under this basic condition, dissociation of a phenolic proton leads to the formation of phenolate ion, which is responsible to reduce the F–C reagent. Upon reduction, the intense yellow colour of F–C reagent turns into a blue colour [22]. The colour change is illustrated by a colour ribbon (Figure 2). 

General methodology: Total phenolic content is determined using Folin–Ciocalteu reagent. To 0.2 µL sample solution (2 mg/mL), 1 mL of F–C reagent and 2 mL of Na_2_CO_3_ were added and mixed carefully. The resulting mixture was brought to 7 mL with deionized water and allowed to incubate at room temperature for 2 h. The absorbance is read at 765 nm. Gallic acid is usually used as a reference standard [23]. 

### Strengths and Limitations


Strengths:
Simple, rapid and reproducible [22]Direct correlation between phenolic compounds and antioxidant activity [24]Can screen many samples in a timely fashion [22]
Limitations:
Non-specific to phenolics [22]



## 7. Free Radical Scavenging Antioxidant Assays

### 7.1. 2,2-Diphenyl-1-picrylhydrazyl Radical Scavenging Capacity (DPPH) Assay

**Figure 3:**
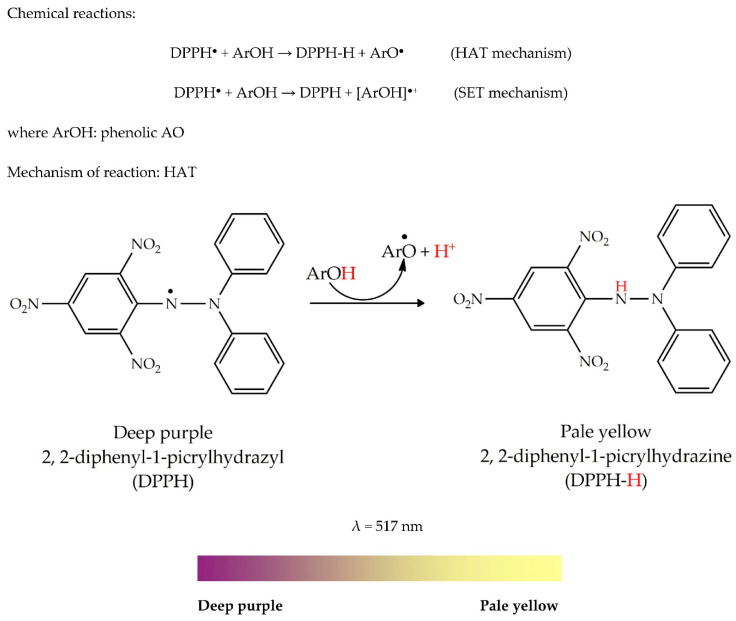
2,2-diphenyl-1-picrylhydrazyl (DPPH) reaction mechanism.

The DPPH^●^ radical was discovered by Goldschmidt and Renn in the 1920s. It was first developed by Blois in 1958 [25,26]. This radical is known for its remarkable stability due to the delocalization of the radical in aromatic rings. It has an intense deep purple colour [27]. In assays, the radical is neutralized by accepting either a hydrogen atom or an electron from an antioxidant species (or reducing agents) during which, it is converted into a reduced form (DPPH or DPPH-H) at the end of the process (Figure 3). The unpaired electron of the DPPH radical absorbs strongly at 517 nm, giving rise to a deep purple colour. However, when an odd electron pairs up with another electron, the initial colour gradually decolorizes into pale yellow. Decolorization is simulated by the colour ribbon below. 

General methodology: First, 50 µL of extract is added to 150 µL methanolic solution of DPPH at 0.1 mM in a 96-well plate. The mixture is then shaken vigorously in the dark at room temperature for 30 min [28]. Results are processed either as an equivalent of a standard reference (Trolox, gallic acid, ascorbic acid, BHA, BHT) or IC_50_. 

#### Strengths and Limitations


Strengths:
Simple, cheap and rapid, since the radical is stable and needs not be generated compared to ABTS [29]Can quantify antioxidants in complex biological systems [29]The radical scavenging time is 30 min, allowing DPPH to react efficiently, even with weak antioxidants [29]Results are reproducible and comparable to other radical scavenging methods [30]Efficient for thermally unstable compounds, since radical scavenging is measured at room temperature [31]Highly sensitive [16]Can screen many samples in a timely fashion [16]Good correlation is usually reported with bioactive compounds (phenols, flavonoids) with a regression factor R > 0.8
Limitations:
DPPH radical chromogens dissolve only organic solvents (lipophilic) [16]DPPH tends to react with other radicals present in the tested samples [29]Since the nitrogen centre is highly sterically hindered by three phenyl groups, DPPH represents a poor model for radical quenching in vivo and in food samples [32]DPPH is sensitive to Lewis bases [33]Upon exposure to light, absorbance of DPPH tends to decrease, which requires analysis in the dark [34]Non-physiological resemblance due to the absence of DPPH free radicals in the human body [11]



### 7.2. Trolox Equivalent Antioxidant Capacity or 2,2′-Azino-bis (3-ethylbenzothiazoline-6-sulfonic acid) (TEAC or ABTS^•+^) Assay

In 1993, Miller and Rice-Evans were the first to report the ABTS^•+^ assay, also known as TEAC [35]. This assay was later improved by Re and Colleagues in 1999 [12]. The improvement was related to how the ABTS^•+^ radical was generated. Compared to DPPH, which is a stable radical by nature, the ABTS^•+^ radical is a radical that should be generated by chemical reactions. Originally, the generation of a radical cation (ABTS^•+^) was done by reacting metmyoglobin (Met-Myb) with hydrogen peroxide (H_2_O_2_) to produce hydroxyl radical (HO^•^). The latter radical causes the reduction of ABTS into its radical in the presence or absence of antioxidants. The reaction is illustrated in Scheme 1. However, this scheme has a major gap. 

For instance, the AO can reduce the HO^•^ radical present in the system together with metmyoglobin and ABTS^•+^, resulting in an overestimation of the antioxidant capacity, which leads to erroneous results. To overcome this problem, an improved method is proposed by eliminating the requirement of HO^•^ radical and metmyoglobin. The improved method generates the ABTS^•+^ radical in only one reaction by reacting ABTS with ammonium or potassium persulfate ((NH_4_)_2_ S_2_O_3_ or K_2_S_2_O_3_, respectively) prior to the addition of AOs. It is important to note that ABTS is in a stoichiometry ratio of 1:0.5 with persulfate salt, meaning that not all ABTSs are oxidized prior to the addition of AO [12,32,36]. The improved reaction is illustrated in Scheme 2. Oxidation of ABTS is a long reaction which takes about 12–16 h. The ABTS^•+^ radical solution is then diluted in ethanol/methanol until an absorbance of 0.7 ± 0.02 is reached at 734 nm. This dilution is done only before the beginning of the assay, as clearly emphasized by Re et al. [12]. 

**Figure 4:**
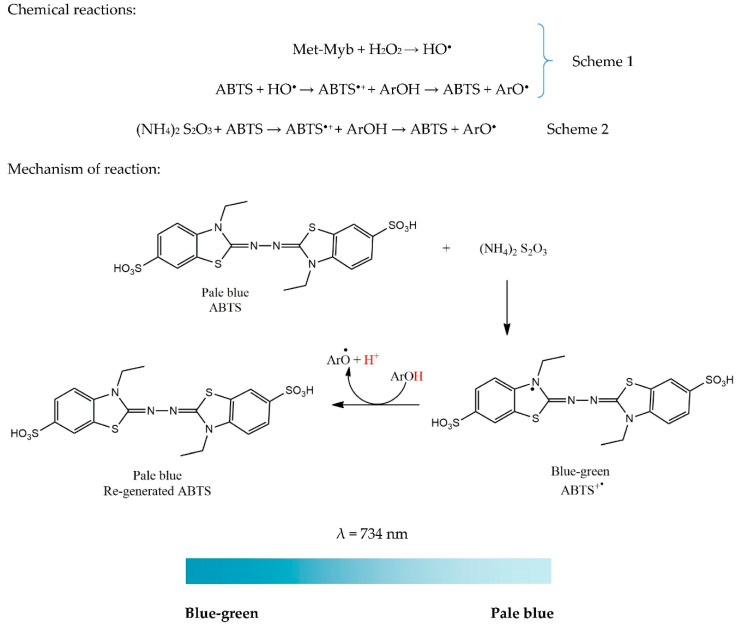
2,2′-azino-bis (3-ethylbenzothiazoline-6-sulfonic acid) (ABTS) reaction mechanism.

Interestingly, the blue-green coloured ABTS^•+^ chromophore may absorb at various wavelengths, namely 645, 734, 815, and 415 nm. However, most investigators have adopted the wavelength of 734 nm because possible interferences are eliminated and sample turbidity is reduced at that wavelength [6,37]. When the ABTS^•+^ radical (unstable form) accepts an electron from the AO, the blue-green colour fades into a pale blue colour, which shows regeneration of ABTS (stable form). Concerning reaction time, existing studies have reported different reaction times, ranging from 1 to 30 min. Re, Pellegrini, Proteggente, Pannala, Yang, and Rice-Evans [12] have stated that completion of an ABTS reaction can be observed after the first minute itself, except for cyanidin and glutathione that show inhibitory activity even after 4 min. The mechanism of the reaction is shown in Figure 4. Decolorization is illustrated with a colour ribbon. 

General methodology: The protocol initially proposed by Re et al. [12] is modified to determine the scavenging capacity against the ABTS^•+^ radical. First, ABTS is dissolved in water to a 7 mM concentration. Then, the ABTS^•+^ radical cation is generated by reacting 7 mM ABTS solution with 2.45 mM potassium persulfate in a ratio of 1:0.5. It is then allowed to stand at room temperature in the dark for 12–16 h. Before starting the assay, the ABTS solution is diluted with ethanol or methanol until an absorbance of 0.7 is reached at 734 nm. To 1 mL of extract to be tested, 2 mL of ABTS solution is added and mixed. The reaction mixture is incubated in the dark at room temperature for 30 min. Results are expressed as equivalent of a standard compound (Trolox, ascorbic acid, gallic acid, BHA, or BHT) for comparison. 

#### Strengths and Limitations


Strengths:
The ABTS cationic radical is soluble in both organic and aqueous media in contrast to the DPPH radical, which dissolves only in organic medium. The ABTS assay can, thus, be used to screen both lipophilic and hydrophilic samples [16]Can be used to determine the antioxidant capacity of numerous compounds, namely carotenoids, phenolic, and plasma [12]The ABTS assay produces reproducible results [6]The ABTS^•+^ radical is stable for more than two days when stored in the dark at ambient temperature compared to DPPH, which has a rather short life, however, Gupta [27] stated that the radical solution is stable for a few months when stored in the refrigerator. We can say that the stability of the ABTS^•+^ radical solution remains debatable [12]Good correlation is usually reported with bioactive compounds (phenols, flavonoids), with generally, a regression factor R > 0.8
Limitations:
The ABTS^•+^ assay is often criticized because the ABTS^•+^ radical does not exist naturally (not found in any biological system) and should be chemically generated. Thus, some literature argued that the ABTS^•+^ radical cannot represent in vivo system [38]Slow reaction for the generation of the ABTS^•+^ radical, which takes about 12–16 h compared to DPPH, which is readily available commerciallyLike DPPH, the ABTS^•+^ radical exhibits high steric hindrance around its nitrogen-centred atom and thus, does not represent a good model for highly reactive radicals, namely OH^•^, NO^•^, O_2_^•−^ or LO(O)^•^, which are present in numerous biological samples [32]



## 8. Thiobarbituric Acid Reactive Species (TBARS) Assay 

The hydroxyl radical (HO^•^) is among the most potent reactive oxygen species (ROS) present in our biological systems. It reacts with polyunsaturated fatty acid moieties that can consequently damage the cell membrane [13]. Among free radicals, OH^•^ is the most harmful ROS which can damage cell membranes and destroy sugar groups and DNA base sequences and even causes cell apoptosis and mutations [39]. The thiobarbituric acid reactive species (TBARS) assay, developed by Kohn and Liversedge in 1944, is a way to measure lipid peroxidation in cells and tissues [40,41]. It was initially used to determine the rate of reaction between HO^•^ and molecules having therapeutic importance. The same methodology is still used to evaluate radical activity between HO^•^ and antioxidants with slight modifications. The protocol consists of many reagents, namely ascorbic acid (AA), deoxyribose, phosphate buffer, ferric chloride, hydrogen peroxide (H_2_O_2_), ethylenediamine tetraacetic acid (EDTA), trichloroacetic acid (TCA), and thiobarbituric acid (TBA). Each one of these reagents has a specific role. The assay is started by complexing EDTA with Fe^2+^ which then reacts with H_2_O_2_ to generate the HO^•^ radical following a Fenton reaction, as shown in Scheme 3 [42]. 

The generation of the radical requires an incubation temperature of 37 °C for a duration of about 12 h. The generated HO^•^ radical then attacks the deoxyribose sugar in the presence of AA to form a mixture of products, as presented in Scheme 4. The purpose of adding AA to the reaction mixture is to increase the rate of deoxyribose degradation by the radical. Heating the resulting mixture of products with TBA in an acidic medium at a low pH will lead to the formation of malondialdehyde (MDA). The formation of MDA can then be detected after its reaction with TBA to form a pink MDA-TBA chromogen [43,44]. 

**Figure 5:**
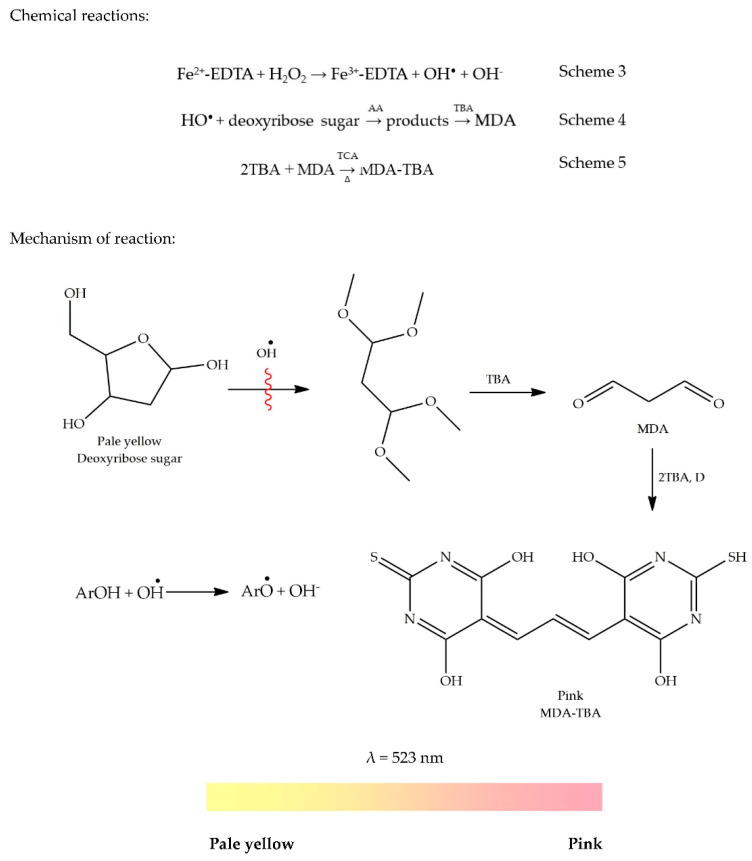
HO^•^ reaction mechanism.

The adduct (TBA)_2_-MDA is formed according to a nucleophilic attack involving a 5-carbon of TBA with 1-carbon MDA followed by dehydration. The same reaction takes place with the second TBA molecule [42]. The proposed mechanism of chromogen formation is shown in Scheme 5. Grotto et al. [42] have stated that to prevent the formation of MDA in an assay, inhibition of deoxyribose degradation is needed. Thus, an AO can be added to the reaction mixture. The scavenging activity toward the HO^•^ radical is measured based on inhibition of deoxyribose degradation. On the same line, we propose the addition of AOs to attack HO^•^ radicals by donating an electron to the latter which can consequently quench the radical. In the absence of the HO^•^ radical, the deoxyribose sugar does not undergo any degradation, thus, hindering the formation of MDA and MDA-TBA adduct, as shown by the red wavy break in Figure 5 In the absence of the MDA-TBA chromogen, the colour of the solution remains pale yellow, indicating good antioxidant activity. The colour change in the absence of an AO is illustrated by a colour ribbon. The mechanism of the reaction is shown in Figure 5. 

General methodology: The hydroxyl radical is generated by mixing 0.28 mL of deoxyribose (10 mM) with 0.41 mL of phosphate buffer (pH 7.4), 0.01 mL of ferric chloride (10 mM), 0.1 mL hydrogen peroxide (10 mM), and 0.1 mL of EDTA (1 mM). Finally, 0.1 mL ascorbic acid (1mM) is added to the premixed reaction mixture containing 0.25 mL sample solution. The resulting mixture is incubated at 37 °C for 12 h. A blank is prepared similarly by mixing 0.25 mL sample solution with 1 mL of the reaction mixture without ferric chloride. Afterwards, 0.75 mL of TCA (2.8%, *w*/*v*) and 0.75 mL of TBA (1%, *w*/*v* in 50 mM NaOH) are added to the incubated sample followed by heating at 100 °C for 1 h. The absorbance of the reaction mixture is measured at 523 nm after the mixture is allowed to cool to room temperature. Results can be expressed as mannitol equivalents (e.g., mg MEs/g extract) [28]. 

**Figure 6:**
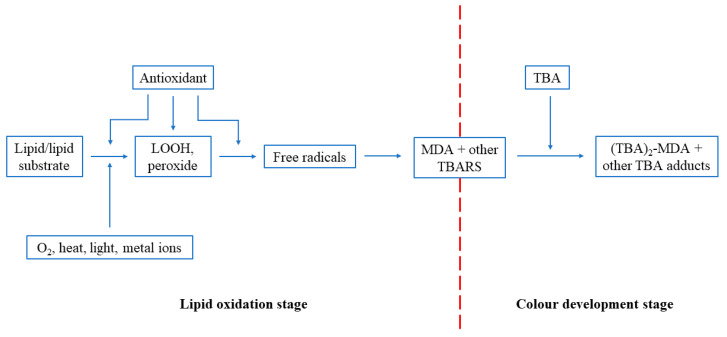
Steps involved in the lipid oxidation and antioxidant action in the TBARS activity assay.

Another way to measure the oxidative damage is by protein and DNA modifications. However, these markers can also be formed by pathways other than from free radicals. Thus, MDA remains the preferred marker to evaluate oxidative damage in tissues and cells. The determination of MDA is possible in numerous biological samples [42]. The production of TBARS occurs nearly at the end of the assay, as shown in Figure 6. This implies that an AO can be introduced into the system at any step of the process prior to the formation of TBARS. Thus, measurement of TBARS gives no indication of the mechanism of action of the antioxidant, i.e., whether it is able to interact with oxygen or metal ions, react directly with hydroperoxides, or intercept the free radicals involved in the breakdown of primary to secondary oxidation products [40]. 

### Strengths and Limitations


Strengths:
Simple, cheap, and accurate results in most cases [42]
Limitations:
Lack of sensitivity and specificity, because TBA reacts with different compounds, namely sugars, amino acids, bilirubin, and albumin [42]MDA is unstable for a long period of time, since it oxidizes into alcohols and acids [42]Aldehydes may also react with TBA, leading to an overestimation of MDA [45]



## 9. Nitric Oxide Radical Scavenging Assay

In the 1980s, the team Furchgott and Zawadzki demonstrated that the endothelium released a substance that can relax blood vessels in response to muscarinic agonists. However, at that time, the chemical nature of this substance was unknown and was, thus, denoted as endothelium-derived relaxing factor (EDRF). Years later, Ignarro and Moncada independently showed that this substance was nitric oxide (NO^•^) due to the chemiluminescent product formed by NO^•^ with ozone. Nitric oxide is a free radical which is not as reactive as other radicals [46]. 

Nitric oxide is generated from amino acid L-arginine found in vascular endothelial cells, specific neuronal cells, and phagocytes by enzymes [47,48]. At low concentrations, NO^•^ plays an effective role in biological activities, namely antimicrobial activity, antitumor effect, vasodilation, and neuronal messenger. However, high levels of NO can cause several health complications, including inflammatory complications such as sclerosis, arthritis, and ulcerative colitis. The toxicity of NO can notably increase upon its reaction with superoxide radical to form a highly reactive anion peroxynitrite anion (ONOO^−^). The latter anion will be discussed later. Many studies have shown that flavonoids can rapidly scavenge NO^•^ radicals [49,50]. 

To measure the NO^•^ radical scavenging activity, diazotization assay or Griess reaction was first developed in 1864 by a German chemist named Johann Peter Griess [51]. The modified experiment involved the reaction of nitrite (NO_2_^−^) with sulfanilic acid (SA) (C_6_H_7_NO_3_S) under an acidic condition, resulting in the formation of a diazonium ion which is subsequently coupled with *N*-(1-naphthyl) ethylenediamine (NED) (C_12_H_14_N_2_) to form a water-soluble and red-coloured azo dye (HO_3_SC_6_H_4_-NN-C_10_H_6_NH_2_) that can be measured at a wavelength of ~540 nm [52,53]. The original method required improvement in order to increase reproducibility, sensitivity, and analysis time. The modified assay shows good results on several occasions. It is now widely used for screening samples to determine their free radical scavenging activities. Interestingly, the same assay is also used to determine nitrite in water in Europe [53]. The assay is started by generating the radical (NO^•^), similar to the HO^•^ radical scavenging assay. The radical is initiated using sodium nitroprusside (SNP; Na_2_[Fe^III^(CN)_5_(NO)]) known to undergo spontaneous degradation in aqueous solution at physiological pH 7.2 to produce NO^•^ (Scheme 6). 

**Figure 7:**
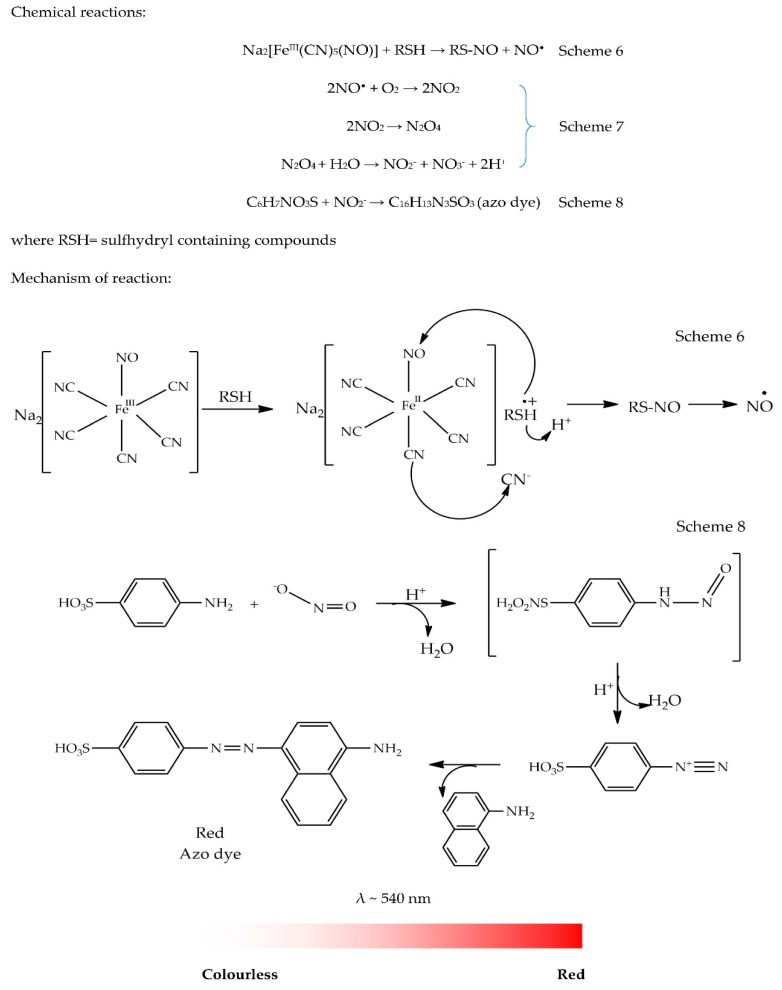
NO^•^ reaction mechanism.

SNP is a non-ferromagnetic species that can be easily reduced to a paramagnetic species, [Fe^II^(CN)_5_(NO)]^3−^, in aqueous solution [54]. Under aerobic conditions, NO^•^ can react with O_2_ to produce nitrate (NO_3_^−^) and nitrite (NO_2_^−^) as stable products that can be quantified using Griess reagent (Scheme 7). The latter reagent is prepared by mixing 1 mL of 0.33% SA with 20% glacial acetic acid. It is allowed to react for 5 min at room temperature. Then, 1 mL of NED is added to the resulting solution to form Griess reagent. The azo group, -N═N-, is the chromophore group of the azo dye compound with a molecular formula of C_16_H_13_N_3_SO_3_. The purpose of adding NED is to increase reproducibility, sensitivity, and solubility of the azo compound in acid and enhance coupling [53]. The absorbance of the chromophore formed can be measured at wavelengths of 546 or 548 nm depending on investigators. The colour change is illustrated with a colour ribbon. However, in the presence of an antioxidant (or absence of NO^•^ radical), formation of NO_3_^−^ and NO_2_^−^ will not occur. Thus, the reaction between NO_2_^−^ and sulfanilic acid will not take place. Consequently, no azo dye compound is formed and no red colour is observed as the solution will remain colourless. The mechanism of the reaction is shown in Figure 7.

General methodology: SNP dissolved in aqueous solution at physiological pH 7.2 can generate nitric oxide whose level can be measured by Griess reaction. Sample solution (0.5 mL) is mixed with SNP (0.5mL, 5mM) in phosphate buffer at pH 7.4 (0.2 M), followed by an incubation at room temperature for 150 min. Similarly, a blank is prepared by adding sample solution in phosphate buffer without SNP. Griess reagent (0.33% sulfanilic acid, 20% glacial acetic acid, 0.1% NED) (1 mL) is added to the incubated sample and allowed to stand for 30 min. Absorbance values of the blank and samples are measured at 548 nm. The absorbance of the blank is then subtracted from that of the sample. Results are expressed either as %inhibition or equivalent of standard compound (e.g., Trolox) [55].

### Strengths and Limitations


Strengths:
Simple, cheap, sensitive, reproducible, and rapid analysis time [53]
Limitations:
This assay may present problems due to rapid scavenging, high reactivity, and swift diffusion [52]



## 10. Peroxynitrite Scavenging Assay

**Figure 8:**
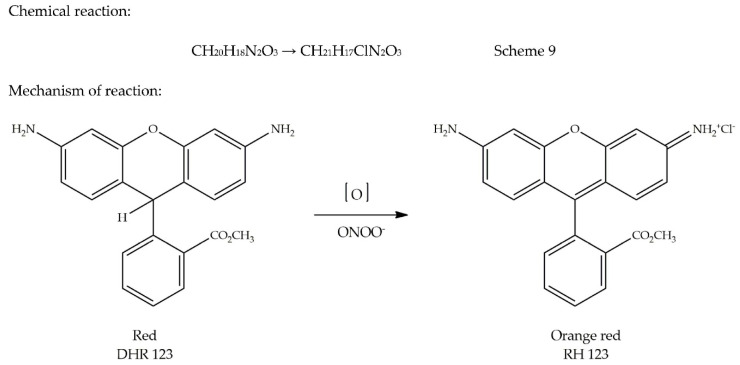
ONOO^−^ reaction mechanism.

Peroxynitrite (ONOO^−^) is a strong oxidant resulting from a fast reaction between nitric oxide and superoxide (O_2_^•−^), which occurs in vascular endothelial cells, Kupffer cells, neutrophils, and macrophages. It was first discovered in 1900 as a biological endogenous oxidant [56]. Peroxynitrite (ONOO^−^) is not a free radical, since unpaired electrons on NO^•^ and O_2_^•−^ can pair up to form a new O-N bond [57]. Although ONOO^−^ is a stable species, its protonation can lead to the formation of a highly reactive acid (ONOOH). The presence of a notable amount of ONOOH can cause numerous problems, including apoptotic cell death, Alzheimer’s disease, atherosclerosis, and rheumatoid arthritis, among others. Since endogenous enzymes for scavenging ONOO^−^ are lacking, there is an urgent need to develop specific ONOO^−^ scavengers [13]. ONOO^−^ scavenging activity is measured by oxidation of dihydrorhodamine 123 (DHR 123; CH_20_H_18_N_2_O_3_) into a fluorescent probe, rhodamine 123 (RH 123; CH_21_H_17_ClN_2_O_3_), at excitation and emission wavelengths of 485 or 505 nm and 529 or 530 nm, respectively, in the presence of an AO [13,58,59]. Therefore, if the AO can successfully scavenge ONOO^−^, no oxidation of DHR 123 will take place. Therefore, no formation of RH 123 will be observed (i.e., reduced formation of orange-red colour). The wavelength at which DHR 123 is measured can explain its red colour in appearance based on a colour wheel (Figure 1). This assay is not commonly used. It lacks information on its origin. The oxidation of DHR 123 is presented in Scheme 9. The mechanism of the reaction is shown in Figure 8.

General methodology: A stock solution of DHR 123 (5 mM) is prepared in dimethylformamide, purged with nitrogen, and kept at −80 °C. Prior to the start of the assay, the stock solution of DHR 123 is diluted to a concentration of 5 µM and placed over ice in the dark. A buffer solution containing 50 mM sodium phosphate (pH 7.4), 90 mM sodium chloride, 5 mM potassium chloride, and 100 µM diethylenetriaminepentaacetic acid (DTPA) was prepared, purged with nitrogen, and placed over ice before use. Scavenging activity of ONOO^−^ by oxidation of DHR 123 is measured fluorometrically at excitation and emission wavelengths of 485 and 530 nm, respectively. The background and final fluorescence intensities are measured at 5 min after treatment without 3-morpholino-sydonimine (SIN-1) or authentic (ONOO^•^). Oxidation of DHR 123 by decomposition of SIN-1 is slowly increased. However, with authentic ONOO^•^, the decomposition is fast, while its final fluorescent intensity is stable with time [13,60]. 

### Strengths and Limitations


Strengths:
Direct physiological resemblance of peroxynitrite (ONOO-) to the human body [61]
Limitations:
Lacks specificity. The rapid decomposition of ONOO^−^ forms NO^•^ and O_2_^•−^. These two species have the potential to oxidize DHR 123. Thus, it can be said that the oxidation of DHR 123 is not directly linked to ONOO^−^ [62]Require expensive equipment and reagent, namely fluorescence spectrophotometer, −80 °C freezer, and DHR 123, which are not easily accessible in all laboratories



## 11. Superoxide Radical Scavenging Assay

The superoxide (O_2_^•−^) radical is formed during a normal respiration process, which reduces 1–3% of the oxygen that we breath into its radical, O_2_^•−^. The reduction of molecular oxygen (O_2_) takes place intracellularly in the mitochondria under normal physiological conditions [63,64,65,66]. The AO enzyme that is responsible for quenching O_2_^•−^ radicals is called superoxide dismutase (SOD). SOD was discovered by McCord and Fridovich in 1969 [67]. This enzyme converts O_2_^•−^ into H_2_O_2_, which is further converted into O_2_ and water by glutathione peroxidase and catalase [44]. The generation of the O_2_^•−^ radical can be done using two systems: (1) a non-enzymatic system involving phenazine methosulphate (PMS; C_13_H_11_N_2_·CH_3_SO_4_), nitroblue tetrazolium (NBT; C_40_H_30_Cl_2_N_10_O_6_), and a reduced form of nicotinamide-adenine-dinucleotide (NADH; C_21_H_27_N_7_O_14_P_2_); or (2) a hypoxanthine-xanthine oxidase superoxide generating system, as described by Robak and Gryglewski [68]. 

**Figure 9:**
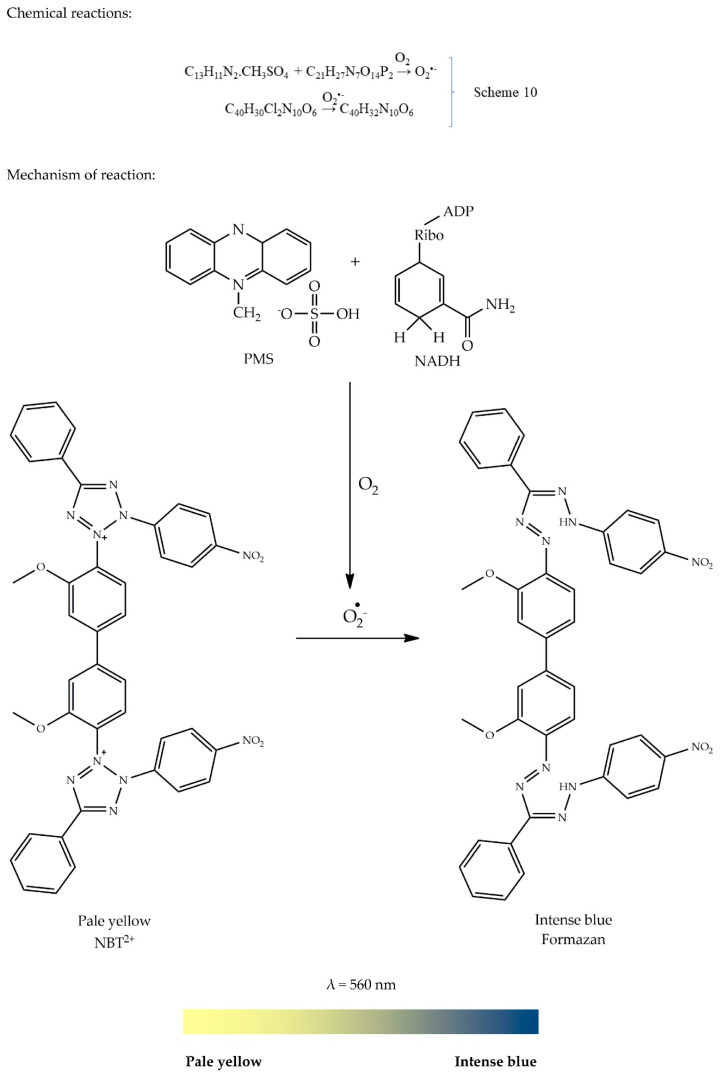
O_2_^•−^ reaction mechanism.

The scavenging activity of AOs towards O_2_^•−^ is assessed in terms of their ability to prevent O_2_^•−^ generation. Prior to the reduction process caused by O_2_^•−^, NBT is a pale-yellow soluble salt. However, upon reduction occurring at a pH of 7.4, the tetrazole ring is disrupted, leading to dismutation which subsequently results in an intense blue insoluble diformazan product (C_40_H_32_N_10_O_6_), as illustrated in Scheme 10 [44,69,70]. Importantly, the addition of a potential AO will react with O_2_^•−^ radical and inhibit the formation of diformazan. Therefore, no intense blue colour will be observed. The colour change in the absence of an AO is illustrated by the colour ribbon in Figure 9. 

General methodology: Sample solution is treated with 0.05 mL phosphate buffer (250 mmol/L), 0.025 mL NADH (2 mmol/L), and 0.025 mL NBT (0.5 mmol/L). The absorbance of the resulting solution is read as a blank at 560 nm. To the resulting solution, 0.025 mL PMS (0.03 mmol/L) is added and allowed to incubate at room temperature for 5 min. The absorbance is read again at the same wavelength. Results are expressed either as %inhibition or equivalent of standard compound (e.g., gallic acid, BHA, ascorbic acid, α-tocopherol, curcumin) [71].

### Strengths and Limitations


Strengths:
O_2_^•−^ is one of the most important radicals produced inside human body. Hence, they bear resemblance to biological systems in contrast to DPPH or ABTS which are synthetic radicals
Limitations:
Non-specific since NBT can be reduced by several reductases apart O_2_^•−^ [69]NBT is an expensive reagent and thus it is not affordable by all laboratories



## 12. Hydrogen Peroxide Scavenging Assay

After the discovery of O_2_ by Lavoisier, Scheele, and Priestley in the 18^th^ century, Thenard was the first one who reported the synthesis of H_2_O_2_ in 1818 [72]. Hydrogen peroxide is a major oxygen metabolite generated in vivo by activated phagocytes and oxidase enzymes. It is a good antimicrobial agent for numerous bacterial and fungal strains [59]. H_2_O_2_ scavenging activity is assessed based on a peroxidase system involving horseradish peroxidase (HRP), which is the most commonly used enzyme in this assay. The assay generally employs the oxidation of scopoletin by an HRP–H_2_O_2_ complex formed upon the addition of H_2_O_2_ and HRP (as illustrated in Scheme 11). Scopoletin is a 7-hydroxy-6-methoxycoumarin found in the roots of plants belonging to the genus *Scopolia*. It is a fluorescent substrate for peroxidase used for the determination of H_2_O_2_. It has a strong blue fluorescence under UV light [73]. The intensity of fluorescence is directly proportional to the concentration of scopoletin or inversely proportional to the concentration of oxidized scopoletin (i.e., H_2_O_2_ concentration) [74]. Therefore, the concentration of H_2_O_2_ can be measured either by monitoring the decrease in fluorescence of scopoletin or by observing the increase in fluorescence caused by the formation of oxidized scopoletin. 

**Figure 10:**
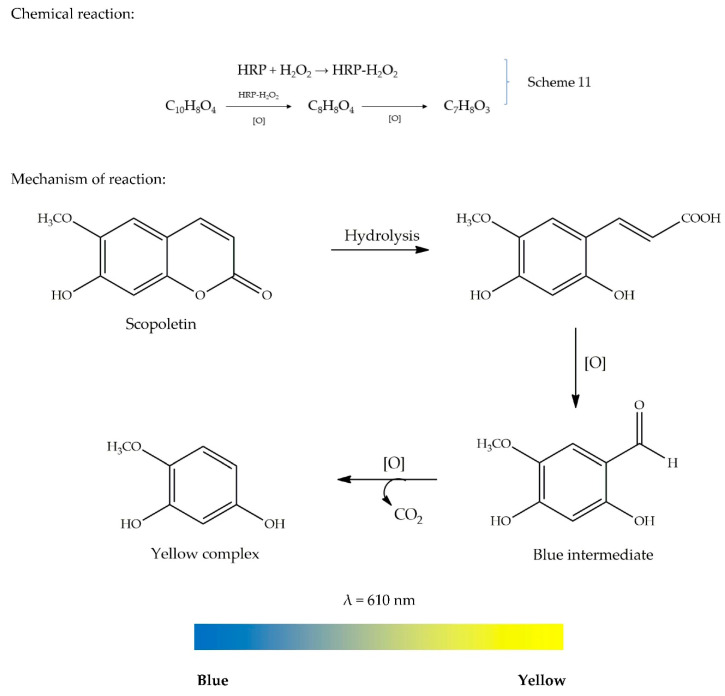
H_2_O_2_ reaction mechanism.

When H_2_O_2_ is mixed with HRP, scopoletin (C_10_H_8_O_4_) is rapidly oxidized by HRP–H_2_O_2_ to form a blue colloidal intermediate product with a chemical formula of C_8_H_8_O_4_, which absorbs light at 560 nm. The formation of this intermediate results from the loss of the blue fluorescence, since scopoletin is being consumed in the reaction. The blue intermediate is slowly oxidized into an insoluble yellow complex (C_7_H_8_O_3_) on standing, which can be read either at 417–402 nm or at 385 nm, depending on different studies [63,75,76]. However, the addition of a potential AO will prevent the oxidation process to occur, thus, hindering the formation of the yellow complex. Consequently, H_2_O_2_ scavenging can be measured [77]. The assay is conducted at pH 4.5. The reaction is stopped with borate buffer (pH 10). After stopping the reaction, the fluorescence or absorbance is then measured. The scavenging capacity may be measured either fluorometrically or spectrophotometrically. The HRP–scopoletin-based method is preferred over the cytochrome *c* peroxidase method, since the latter method requires complex instrumentation [78]. There is not much information published on the mechanism of reaction on this assay. However, we proposed one mechanism, as shown in Figure 10, together with the colour ribbon representing the colour change in the absence of an AO.

General methodology: Sample solution (100 µL: 0.05 mg/mL) is added to 100 µL of 0.002% of hydrogen peroxide. To the resulting solution, 0.8 mL of phosphate buffer (0.1 M) and 100 mM sodium chloride are added. The reaction mixture is allowed to incubate at 37 °C for 10 min. After the incubation period, 1 mL of phenol red (0.2 mg/mL) with 0.1 mg/mL HRP in 0.1 M phosphate buffer are added. After incubation for 15 min, 50 µL sodium hydroxide (1 M) is added and the absorbance is measured at 610 nm. The hydrogen peroxide activity can be expressed as %inhibition [79].

### Strengths and Limitations


Strengths:
Easy and sensitive [59]Specific [80]Can quantify H_2_O_2_ level in cells [78]Reagents are easily available
Limitations:
Substrates are pH sensitive [80]Several reductants/or antioxidants for e.g., thiols, ascorbate can compete with scopoletin, leading to an underestimation of H_2_O_2_ formation [80]Similarly, catalase released from disruption of cells may compete with HRP for H_2_O_2_ [80]Quartz cuvettes should be used which are expensive and not affordable by all laboratories



## 13. Reducing Potential Antioxidant Assays

As discussed earlier, antioxidants can stabilize radicals by donating electrons. In this type of AO assay, the mechanism involves the reduction potential of transition metals, namely iron (Fe) and copper (Cu). It is acknowledged that there is a major uncertainty concerning the role of AO towards free radicals in the presence of metal ions, namely Cu (II) and Fe (III). The role of AO is uncertain in the presence of these metal ions because it is still unclear whether the AO will scavenge the free radicals or chelate with metal ions [81]. Additionally, working with coloured radicals can be problematic, since it is difficult to generate them. In addition, it is hard to maintain their stability (e.g., ABTS or DPPH) [82]. The antioxidant capacity of a biological sample cannot be evaluated by a single assay, since many factors are not taken into consideration. For instance, not all methods can be used to screen both lipophilic and hydrophilic samples. Possible interferences in reaction mixtures can lead to underestimation or overestimation. We will discuss each reducing potential assay in greater detail in the next sections. 

### 13.1. Ferric Ion Reducing Antioxidant Power (FRAP)

**Figure 11:**
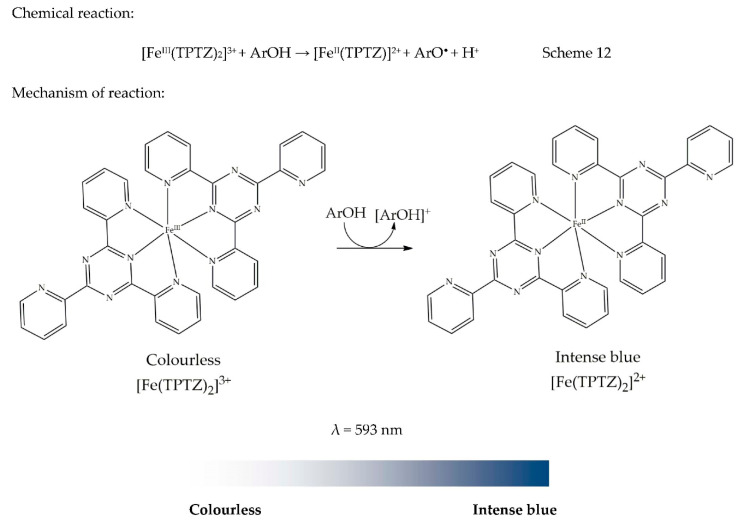
Ferric reducing antioxidant power (FRAP) reaction mechanism.

The ferric ion reducing antioxidant power or ferric reducing ability of plasma, abbreviated as FRAP, was developed by Iris Benzie and J. J. Strain [83]. The FRAP method is based on the reduction of ferric-tripyridyltriazine [Fe^III^(TPTZ)]^3+^, forming an intense blue coloured ferrous complex [Fe^II^(TPTZ)]^2+^ under acidic conditions (pH 3.6). The chemical reaction is presented in Scheme 12. The colour developed in this assay is intense blue, which is the complementary colour of orange, as shown in the colour wheel in Figure 1. This explains why the absorbance is read at 593 nm. This method was developed in such a way that it could be conducted in every laboratory due to its simplicity, high reproducibility, and simple instrumentation [83]. The redox potential of Fe (III) is approximately 0.70V, which is comparable to the redox potential of ABTS^•+^ (0.68 V) according to the review compiled by Huang et al. (2005). Interestingly, there is a thin line between ABTS^•+^ and FRAP methods except that the ABTS assay is conducted at neutral pH while FRAP is carried out under acidic conditions [8]. However, this assay is non-specific. This is because if any species present in the reaction mixture possesses a redox potential lower than that of Fe (III) (<0.70 V), that species will be responsible for the reduction in [Fe^III^(TPTZ)_2_]^3+^ [83], leading to an underestimation. Importantly, while preparing FRAP reagent, it is essential to add reagents in a specific order. For instance, acetate buffer is added first. FeCl_3_ is then added, while TPTZ is added last. This order is vital to prevent the reduction of FeCl_3_ by TPTZ. Colour change and mechanism of reaction are presented in Figure 11. 

General methodology: FRAP reagent is prepared by mixing 0.3 M acetate buffer (pH 3.6) and 10 mM 2,4,6-tris(2-pyridyl)-S-triazine in 40 mM hydrochloric acid and ferric chloride (20 mM) at a ratio of 10:1:1 (*v*/*v*/*v*). The sample solution (0.1 mL) is added to 2 mL premixed FRAP reagent and allowed to incubate at room temperature for 30 min. The sample absorbance is then read at 593 nm. FRAP activity can be expressed as equivalents of Trolox, gallic acid, ascorbic acid, quercetin, or α-tocopherol [28].

#### Strengths and Limitations


Strengths:
Simple and inexpensive instrumentation [84]Highly reproducible and sensitive [83]Can screen a wide spectrum of biological samples including plasma, blood, serum, saliva, tears, urine, cerebrospinal fluid, exudates, transudates, and aqueous and organic extracts of drugs, foods, and plants [85]Good correlation is usually observed with H_2_O_2_ scavenging assay [86]
Limitations:
Non-specific [83]



### 13.2. Cupric Reducing Antioxidant Capacity (CUPRAC) 

**Figure 12:**
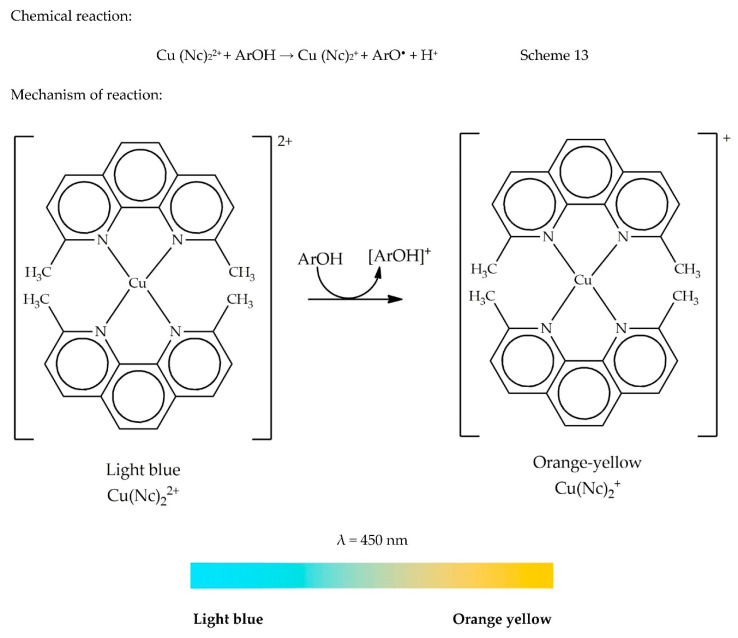
Cupric reducing antioxidant capacity (CUPRAC) reaction mechanism.

The CUPRAC method was developed by Apak, Guclu, Ozyurek, and Karademir [82] from the Analytical Chemistry Department of Istanbul University, seven years after the FRAP method was developed. The sole reason for developing this assay was to bring forward a method that could express the ‘total antioxidant’ as a nutritional index for food labelling due to the lack of a standard quantitation method. Indeed, this method has been proven to be effective for many polyphenols (namely, phenolic acids, hydroxycinnamic acids, flavonoids, carotenoids, and anthocyanins) in addition to thiols, synthetic AOs, and vitamins C and E. It has been used by many investigators in different laboratories over the last few years since its development. The chromogen used in this assay is bis(neocuproine) copper (II) cation [Cu (Nc)_2_^2+^]. Upon its reduction by an AO, the light blue chromophore is reduced into an orange-yellow bis(neocuproine) copper (I) chelate [Cu (Nc)_2_^+^] that can be read at 450 nm. Reaction time to reach the completion may vary between 30 and 60 min, depending on how fast the AO is. According to a comprehensive review compiled by Özyürek et al. [87], it is essential to allow completion of CUPRAC reaction. If an AO develops colour at a slow pace, an incubation at 50 °C in a water bath for 20 min may be needed, which is viewed as a limitation. While conducting the CUPRAC assay, a sample blank without copper (II) chloride (CuCl_2_) should be prepared, since absorbance is measured between 400 and 500 nm. However, in the FRAP assay, no sample blank is required, since the absorbance falls after 500 nm. The mechanism of the reaction and the colour change are illustrated by the colour ribbon shown in Figure 12. The chemical reaction is presented in Scheme 13. 

General methodology: A reaction mixture containing CuCl_2_ (1 mL, 10 mM), neocuproine (1 mL, 7.5 mM), and ammonium acetate aqueous buffer at pH 7 (1 mL, 1 M) is prepared. The sample solution (0.5 mL) is added to the premixed reaction mixture. A blank is prepared in a similar manner by adding a 0.5 mL sample solution to 3 mL premixed reaction mixture without CuCl_2_. The sample and the blank are allowed to incubate at room temperature for 30 min. Their absorbance values are then read at 450 nm. CUPRAC capacity can be expressed either as %inhibition or equivalent of a standard compound, namely Trolox, gallic acid, ascorbic acid, quercetin, or α-tocopherol [28].

#### Strengths and Limitations


Strengths:
Reagents are cheap, relatively stable, and more accessible than DPPH and ABTS reagents [81]Rapid colour development [81]Can screen both lipophilic and hydrophilic samples [81]The assay is performed at pH 7, which is close to the physiological pH in contrast to the unrealistic acidic pH 3.6 of FRAP [81]Effective for evaluating the AO capacity of synthetic mixtures [81]It can detect glutathione and thiol-type AO in contrary to FRAP. The reason lies behind the fact that Fe (III) has a half-filled d orbital which contribute to its chemical inertness, while the electronic structure of Cu (II) triggers fast kinetics [87]Simple instrumentation requiredNo interferences from chemicals found in solutions reported yetShows good correlation with numerous polyphenolics namely flavonoids, phenolic acids and also, with other AO methods, namely ABTSA linear correlation is usually observed with the phosphomolybdenum assay
Limitations:
Unable to measure antioxidant enzymes [81]Depending on CUPRAC version, longer times of measurement may be required [81]Sometimes requires incubation at 50 °C in a water bath for 20 min for compounds which develop color slowly namely naringin and naringenin [82]



## 14. Potassium Ferricyanide Assay

The potassium ferricyanide assay is another type of FRAP method based on the reduction of Fe (III) to Fe (II), usually abbreviated as PFRAP. It can be used to measure the reducing capacity of AO. In addition, it has several other usages such as for the determination of reducing sugars in plants [88], the estimation of pravastatin sodium [89], the determination of dopamine hydrochloride in serum or pharmaceutical samples [90], and the detection of catalase isozymes in either plants or animals [91]. It is noteworthy to point out that this assay can form different coloured complexes upon reaction of the sample of interest with potassium ferricyanide-Fe (III). For instance, in the determination of pravastatin sodium drug, a green chromogen is formed, which exhibits a maximum absorption at 737 nm [89]. However, in the determination of dopamine hydrochloride, a Prussian blue complex is formed, exhibiting a maximal absorption at 735 nm [90]. Thus, it can be said that the colour of the chromogen formed in this AO assay depends on the sample under investigation. In this review, we are interested in biological samples derived from plant materials. Thus, the methodology, colour ribbon, and principle are focused on for the formation of a Prussian blue complex. 

The potassium ferricyanide (K_4_[Fe (CN)_6_]^3−^) assay was originally developed to study the rate of sugar oxidation by Ariyama and Shaffer in 1928. Later, it was implemented in blood sugar analysis [92]. After this, the authors in [93] used this method for the determination of reducing sugars present in plants. Potassium ferricyanide with chemical formula K_4_[Fe (CN)_6_]^3−^ is a bright red salt that contains octahedrally coordinated ion [Fe (CN)_6_]^3−^. 

**Figure 13:**
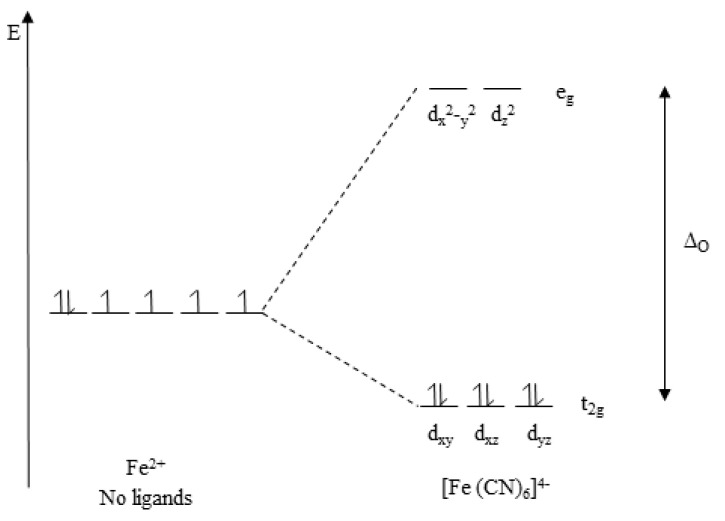
Fe (II) complexes have six electrons in the 5-d orbitals. In the absence of a crystal field (ligands), the orbitals are degenerate. In the presence of ligands (CN^−^), the d-orbitals split into e_g_ and t_2g_ orbitals with an energy difference of ∆_O_.

As highlighted in Section 3.1, the colour of a complex results from a change in its electronic transitions. Before explaining the important theory behind the colour change of potassium ferricyanide-Fe (III), some basic chemistry knowledge will be provided. To start with, we need to understand that coordination compounds (often called complexes) are molecules that contain covalent bonds between a transition metal ion and one or more ligands. Such coordinate covalent bonds are formed when the metal ion acts as a Lewis acid (electron-pair acceptor) and ligands act as Lewis bases (electron-pair donors). The covalent bond is formed when the molecular orbital consisting of the lone pair of electrons present on ligands overlap with d-orbitals of the metal ion. These d-orbitals are the frontier orbitals of transition metal complexes. Numerous physical properties of coordination compounds (or complexes), such as colour, shape, reactivity, and stability, are related to the electron occupancy of d-orbitals of the metal ion. Crystal Field Theory (CFT) is the simplest model to explain the structure and properties of transition metal complexes. CFT focuses on the interaction of five d-orbitals of the transition metal ion with ligands surrounding it. According to CFT, an octahedral complex is formed due to electrostatic interaction of the transition metal ion with six negatively charged ligands. To clearly understand CFT, we should know that in the absence of ligands, these five d-orbitals are at the same energy level (degenerate). However, when the metal ion is in the proximity of ligands, those d-orbitals will split into two groups of different energy levels, namely e_g_ and t_2g_, due to repulsion caused by the ligands. These e_g_ orbitals now have two orbitals (d_x_^2^ − _y_^2^, d_z_^2^) at a higher energy level than the other three t_2g_ orbitals (d_xy_, d_xz_, d_yz_). The energy difference between the e_g_ and t_2g_ orbitals is denoted as ∆_O_, as illustrated in Figure 13. The energy difference depends on the nature of the ligands. In the case of potassium ferricyanide, cyanide (CN^−^) is considered as a strong field ligand that causes a big splitting. The value ∆_O_ increases as the oxidation state of the metal ion increases. In this assay, Fe (III) is reduced to Fe (II). Thus, ∆_O_ is decreased. Energy (∆_O_) is related to wavelength with the following equation: E (∆_O_) = hc/λ, where h is Planck’s constant and c is the speed of light [94]. 

The reduced Fe (II) is read at 700 nm, meaning that a photon at wavelength of 700 nm is absorbed, promoting an electron from a t_2g_ to an e_g_ orbital, which makes the Fe (II) enter an excitation state (unstable). When the electron returns to its ground state (stable), the energy emitted is equal to the energy corresponding to the red region in a colour wheel. This explains why the reduced form of potassium ferricyanide-Fe (II) appears Prussian blue (dark blue) in colour. 

**Figure 14:**
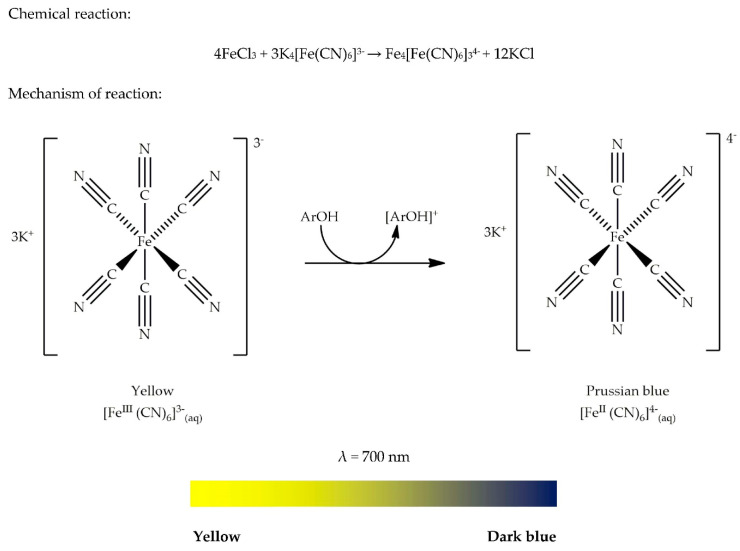
Potassium ferricyanide reaction mechanism.

The purpose of heating the resulting solution containing sample, buffer, and potassium ferricyanide at 50 °C for 20 min, as proposed in the general methodology of this assay, is to reduce the maximum amount of potassium ferricyanide [88]. The mechanism of the reaction and the colour change are illustrated by the colour ribbon, as shown in Figure 14. 

General methodology: Briefly, sample solution (0.5 mL) is mixed with 0.5 mL phosphate buffer (0.2 M, pH 6.6) and 0.5 mL potassium ferricyanide (1%). The resulting mixture is incubated at 50 °C for 20 min. After the incubation period, 0.5 mL trichloroacetic acid (10%), 2.5 mL deionized water, and 0.5 mL ferric chloride (0.1%) are added to the mixture. The sample absorbance is read at 700 nm. The reduction of Fe (III) to Fe (II) can be expressed as %inhibition or equivalent of a standard compound (e.g., Trolox) [28].

### Strengths and Limitations


Strengths:
Cheap, simple, and reliable method [88]Since absorbance is read at a high wavelength, possible interference from the reaction mixture is minimized [90]Simple instrumentation is used
Limitations:
If the samples are very active, pellets can be observed in tubes. Thus, centrifugation will be neededThe assay cannot be performed in microplates which can be time-consuming and tiring while screening many samplesIf samples have a high level of protein, precipitation with trichloroacetic acid may be hard



## 15. Phosphomolybdenum Assay

The phosphomolybdenum assay is a quantitative method developed by Prieto et al. [95]. This assay was originally used to quantify vitamin E in seeds. Considering simplicity and sensitivity, its application has been extended to plant extracts [95]. The assay is widely used by many investigators. The phosphomolybdenum method follows either an ET or HAT mechanism, causing the reduction of molybdenum (VI) to molybdenum (V). The absorbance of the greenish-blue complex can be read at 695 nm [96]. The chemical reaction is presented in Scheme 14. 

A reaction endpoint of about 90 min is enough for complete formation of the greenish-blue phosphomolybdenum complex. The greenish-blue colour results from the reduction of ammonium molybdate into an oxide known as the Keggin ion [H_3_PO_4_(MoO_3_)_12_] under acidic conditions. The resulting Keggin ion is then reduced into [H_4_PMo_8_^VI^Mo_4_^V^O_40_]^3−^ in the presence of an AO [97,98]. 

The formation of the phosphomolybdenum complex is possible at room temperature. However, the rate of reaction is very slow, and the yield of the complex is low. Generally, to increase the rate of a chemical reaction, we either increase the concentration, temperature, or the surface area of the reactant or by adding a catalyst to the reaction. In the case of phosphomolybdenum assay, Prieto, Pineda, and Aguilar [95] have reported that the formation of the complex is temperature-dependent. Indeed, an increase in temperature of 95 °C has accelerated the reaction rate and increased the yield of the green phosphomolybdenum complex [95]. To determine the precision of this assay, a comparison was made with a standard HPLC method. Statistical results from Student’s *t*-test showed no significant difference between the phosphomolybdenum assay and the HPLC method at a confidence level of 95% [95]. 

**Figure 15:**
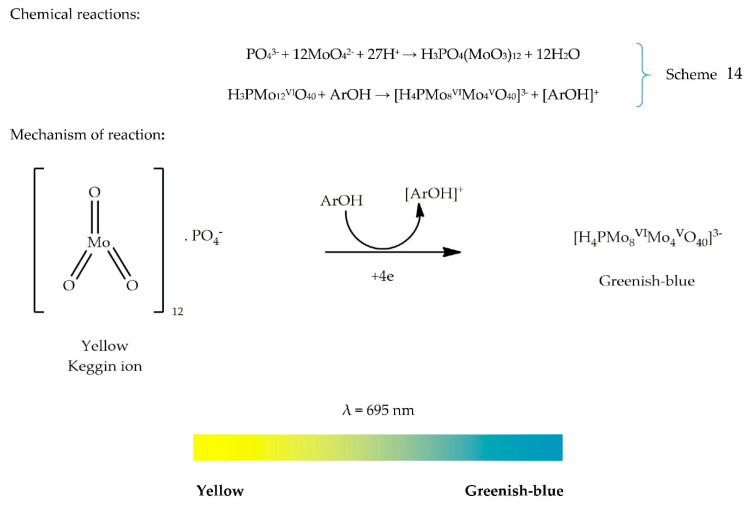
Phosphomolybdenum reaction mechanism.

The colour developed in this assay is green or greenish-blue, which is the complementary color of red, as shown in the colour wheel (Figure 1). This explains why the absorbance is read at 695 nm. Furthermore, this assay does not require any blank since the maximum absorbance is read after 500 nm, similar to the FRAP assay. The phosphomolybdenum assay has many pros and cons as all other AO assays. One of its main limitations is for screening essential oil samples. For instance, if an essential oil sample does not show good DPPH activity, significant TAC can be observed with the phosphomolybdenum assay, showing pronounced absorbance values which then require dilution of the sample. The reason behind this inconsistency is related to complex chemical compositions of essential oils. Essential oils are complex biological samples containing multiple compounds, mainly terpenoids, thymol, carvacrol, and menthol that are strong reducing agents. Interestingly, the correlation between phosphomolybdenum assay and other AO assays remains debatable, since some studies have reported a correlation between phosphomolybdenum assay and free radical scavenging activity [96], while other studies have denied such correlation [99]. Similarly, one study has suggested a good correlation between phosphomolybdenum assay and reducing capacity assays (FRAP, CUPRAC) [100], while another study has reported a weak correlation between FRAP and CUPRAC assays [101]. Prieto, Pineda, and Aguilar [95] have highlighted that DPPH and ABTS^•+^ can detect AOs such as flavonoids and phenols, while phosphomolybdenum can generally detect AOs, namely ascorbic acid, some phenolics, α-tocopherol, and carotenoids. This may explain why a high polyphenolic content does not reflect a significant TAC in the phosphomolybdenum assay. The colour change and mechanism of reaction are presented in Figure 15. 

General methodology: Phosphomolybdenum reagent solution is prepared by mixing 0.6 M sulfuric acid, 28 mM sodium phosphate, and 4 mM ammonium molybdate. The sample solution (0.3 mL) is added to the premixed phosphomolybdenum reagent solution and allowed to incubate at 95 °C for 90 min. The sample absorbance is read at 695 nm. Results can be expressed either as %inhibition or equivalent of a standard compound, namely ascorbic acid [28,95]. 

### Strengths and Limitations


Strengths:
Formation of the phosphomolybdenum complex is independent of the different organic solvents (hexane, methanol, ethanol, and dimethyl sulfoxide) used to prepare AO or extract stock solution [95]Simple, sensitive, and cheap reagents are used [95]Can screen a wide spectrum of samples, including lipophilic plant extracts, vegetal oils, butter, serum, pharmaceutical, and cosmeceutical samples [95]
Limitations:
Bad correlation is observed with bioactive compounds (phenolics, flavonoids) [86]A weak correlation is usually reported with free radical scavenging assays, namely DPPH [99]Time-consuming. Since high temperature (95 °C) is required, the assay should be performed in test tubes instead of microplates, which could be problematic while screening a large number of samplesBad correlation with bioactive compounds in essential oil samples due to their complex chemical compositionNon-specific, since the assay does not detect only phenolics but also ascorbic acid, carotenoids and α-tocopherol [95]



## 16. Metal Chelating (Ferrous Ion Chelating) Assay

Free radicals can also originate from heavy and transition metals, namely mercury, lead, arsenic, and iron, leading to diseases associated with oxidative stress. To eliminate these noxious metals, chelation therapy is applied. Unfailingly, AOs once again have proven their worth in radical chemistry. One of the possible mechanisms followed by AOs is chelation of transition metals [102]. Basically, chelation therapy is a treatment that uses medicine (drugs) to remove these toxic metals from our body via excretion (urine). Arsenic is one of the oldest poisonous agents known. Long exposure to arsenic may lead to numerous health complications, including neurodegenerative diseases, cardiovascular diseases, and skin cancer [103]. Another metal ion involved in chelation therapy is iron. Although iron takes part in every cell function, it is frequently responsible for several pathological diseases, namely liver and heart problems, cancer, neurodegenerative diseases, and diabetes. Treatment of iron toxicity involves decontamination of gastrointestinal and administration of chelating agents [104]. The removal of iron, copper, and lead from the central nervous system is a slow process, since penetration of chelators across the blood–brain barrier is restricted [103]. To improve the efficacy of chelation therapy, Flora and Pachauri [104] have suggested the use of a combination therapy consisting of more than one chelating agent or the use of AOs or nutraceuticals. 

**Figure 16:**
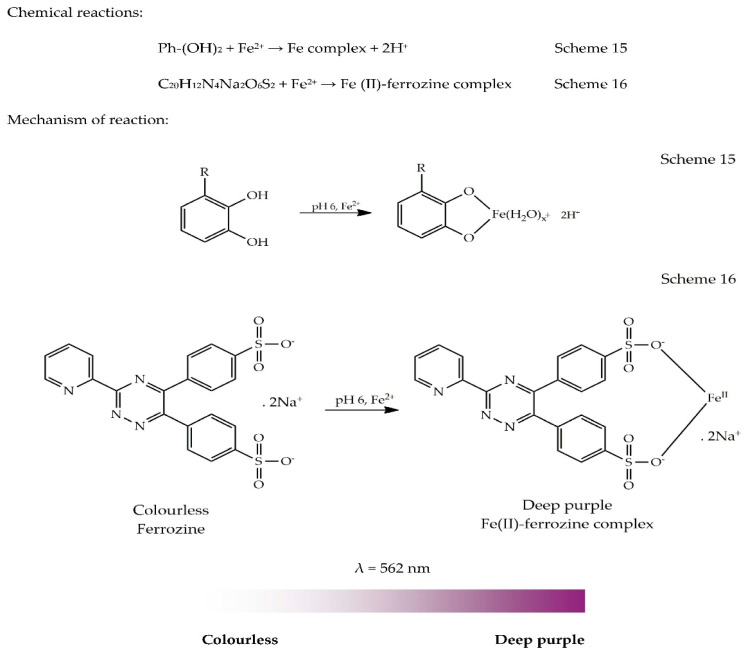
Metal chelating reaction mechanism.

The ferrous ion chelating assay is performed according to the method described by Dinis et al. [105]. Under mild acidic conditions (pH 6), phenolic compounds (Ph-OH) can only bind with a fraction of Fe^2+^, while the remaining Fe^2+^ ions can react with ferrozine (C_20_H_12_N_4_Na_2_O_6_S_2_) to form a ferrous ion–ferrozine complex which is stable, water-soluble and red or deep purple in colour. The chemical reaction is presented in Scheme 15. In the presence of a chelator/extract/AO, the formation of the complex is hindered, leading to a loss in the red or deep purple colour. The chemical reaction is presented in Scheme 16. Measurement of this decrease in colour by spectrophotometry gives an estimation of the binding ability of the chelator/extract/AO. The ferrous ion–ferrozine complex shows maximum absorbance at 562 nm. The higher the absorbance value at 562 nm, the higher the concentration of the ferrous ion–ferrozine complex and the lower the binding ability of the chelator/extract/AO [17,106]. The mechanism of the reaction and the colour change are illustrated by the colour ribbon in the absence of an AO, as shown in Figure 16. 

General methodology: Briefly, sample solution (2 mL) is added to 0.05 mL FeCl_2_ solution (2 mM). The reaction is initiated by adding 0.2 mL ferrozine (5 mM). A blank is prepared without ferrozine (i.e., to 2 mL sample solution, 0.05 mL FeCl_2_ (2 mM), and 0.2 mL water are added). The reaction mixture is incubated at room temperature for 10 min. The sample and blank absorbances are read at 562 nm. The absorbance of the blank is subtracted from that of the sample to obtain real absorbance. Results are expressed as either %inhibition or an equivalent of a standard compound. EDTA or BHA can be used a positive control [28,105].

### Strengths and Limitations


Strengths:
Easily available and cheap reagents [17]Simple instrumentation is usedGood repeatability and reproducibility [17]Assay can be carried out in both test tubes and 96-well microplates
Limitations:
Non-specific, since this assay does not only react with phenolic compounds but also with peptides and sulphates present in the mediumSometimes, results from total bioactive components (phenolic, flavonoids) assay do not correlate with the results of metal chelatingBad correlation with FRAP, CUPRAC, ABTS, and DPPH assays



## 17. β-Carotene Bleaching Assay

**Figure 17:**
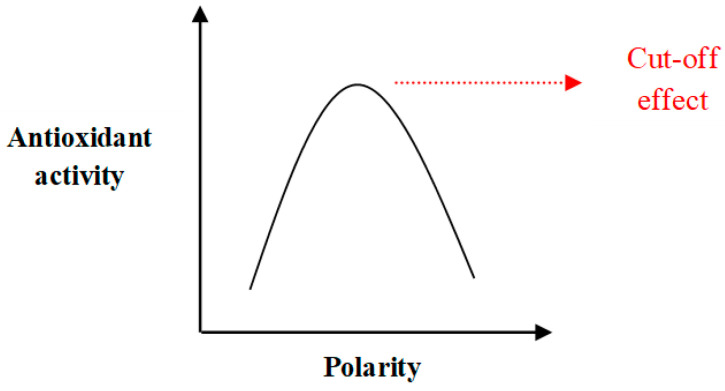
Cut-off effect of antioxidant activity with respect to polarity (Adapted from Shahidi and Zhong [109]).

Antioxidant capacity determined by the β-carotene bleaching assay involves a different scenario compared to the other AO assays discussed above. This assay measures the rate of oxidative destruction of β-carotene by free radicals generated from oxidized linoleic acid in an emulsion system. In the presence of an AO, bleaching of β-carotene occurs [107]. Since an emulsion system is involved in this AO method, the famous theory called ‘Polar paradox theory’ is directly applied. William. L. Porter introduced this theory in 1980. According to this theory, polar (hydrophilic) AOs are more effective in less polar media (bulk oils) while non-polar (lipophilic) AOs are more effective in more polar media (emulsions), solely based on the different polarities in which AOs are present [108]. However, the authors in [109] have suggested that this theory should be re-evaluated, since the theory suffers some contradictions based on several studies conducted. For instance, in addition to the polarity of media, molecular size, and concentration of AOs should be taken into consideration. A non-linear relationship (a bell-shaped curve) between AO activity and polarity has been observed due to interference of the molecular size of AO in this relationship, as illustrated in Figure 17. This curve explains that as the size of chain lengths of phenolic AOs increases, the lipophilicity also increases, resulting in an increase in AO activity until a threshold is reached. After a certain chain length, a drastic drop in AO activity is observed. This threshold phenomenon is referred to as the cut-off effect. Reasons behind such cut-off effect have been discussed in detail in the review of Shahidi and Zhong [109].

**Figure 18:**
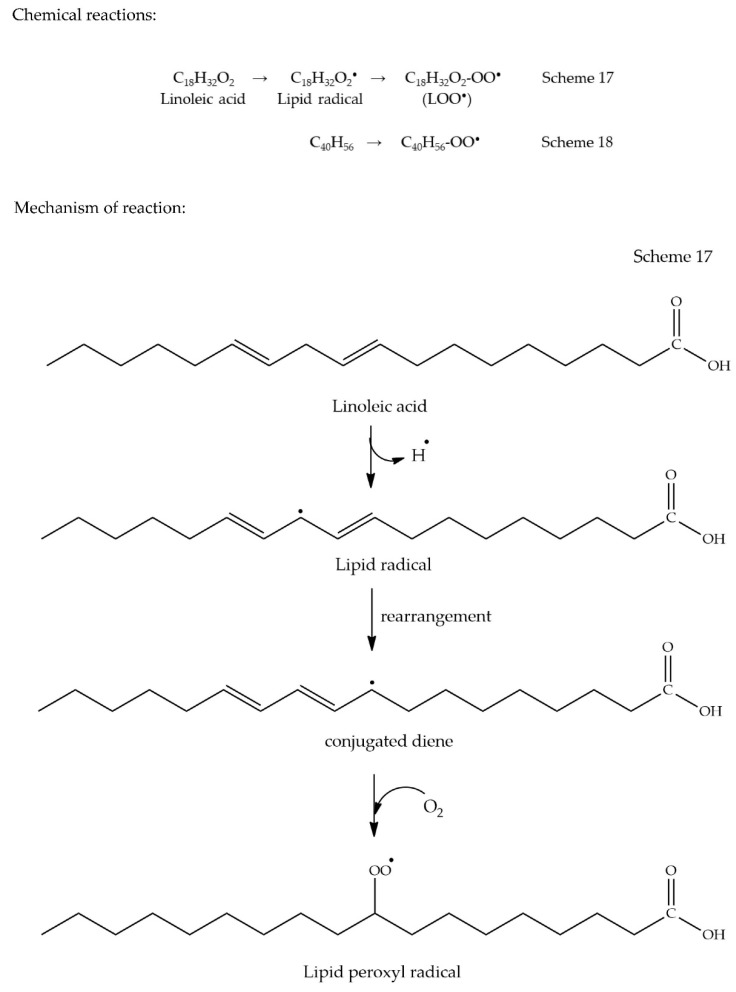

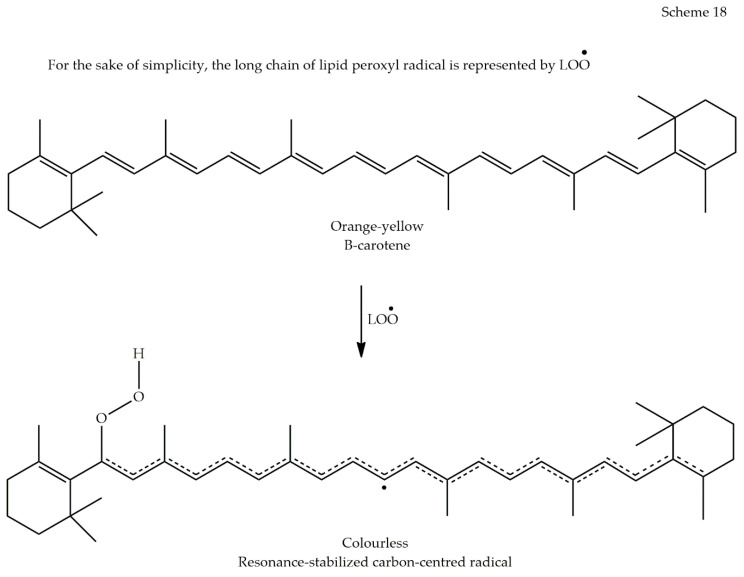
β-carotene reaction mechanism.

Based on the polar paradox theory, derivatives with high lipophilicity exhibit a stronger inhibitory effect on β-carotene bleaching. It is common to observe a high surface-to-volume ratio of oil-in-water emulsions in food systems. Consequently, it is important to determine the effectiveness of AOs in oil-in-water emulsions to evaluate their AO properties before their application in food systems [110]. The prevention of autooxidation of emulsified linoleic acid in extracts was first evaluated using the method developed by Marco [111]. The protocol was modified over the years for convenience while minimizing its weakness. The modified method is highly reproducible compared to the original one. It can quantify both AOs and pro-oxidants. Although the modified method has been accepted as a method for screening various food and biological samples, it is not considered as a universal method [112]. According to published literature, the level of reproducibility of this assay differed. For instance, Prieto et al. [112] reported high reproducibility, while Mikami et al. [113] defined the rate of reproducibility as low. The β-carotene–linoleic acid method involves decolorization of the orange-yellow or dark yellow β-carotene solution caused by radicals generated (lipid or lipid peroxyl radicals) by instantaneous oxidation of fatty acids present in an aqueous emulsion of linoleic acid (C_18_H_32_O_2_) and β-carotene (C_40_H_56_) (lipophilic oxidizable substrate). Lipids or lipid peroxyl radicals are generated from linoleic acid upon exposure to oxygenated distilled water. Once lipids or lipid peroxyl radicals are formed, they are attacked by β-carotene, causing decolorization of β-carotene. The decolorization is due to the breaking of π-conjugation by the addition reaction of radicals into a C=C bond of β-carotene. Chemical reactions are presented in Schemes 17 and 18. In the presence of an AO, the decolorization is slowed down because AO will compete with β-carotene to quench radicals. Quantification of the AO is based on the different rates that β-carotene decays, usually measured at 490–470 nm [112,114,115]. The mechanism of the reaction and the colour change are illustrated by the colour ribbon in the absence of an AO, as shown in Figure 18. 

General methodology: The β-carotene–linoleic acid model system is prepared by dissolving 0.5 mg β-carotene in 1 mL chloroform followed by the addition of 25 mL linoleic acid and 200 mg Tween 40. A rotary evaporator is used to completely evaporate the chloroform added into the system. To the resulting solution, 100 mL of oxygenated distilled water is added followed by vigorous shaking. To the test tube containing 0.5 mL sample solution (1 mg/mL), 1.5 mL of the reaction mixture is added. The emulsion system is then incubated at 50 °C for 2 h. Blank and standard solutions are similarly prepared. Absorbance values of the sample and the blank are then read at 490 nm. The measurement of the absorbance is continued until the colour of β-carotene disappears [28,112]. The bleaching rate (R) of β-carotene is calculated using Equation (1):

(1) $$R=\left[\frac{ln\left(a|b\right)}{t}\right]$$

where *ln* = natural log, *a* = absorbance at time 0, *b* = absorbance at time *t* (30, 60, 90, and 120 min). The antioxidant activity (*AA*) is calculated in terms of % inhibition with respect to the control using Equation (2):

(2) $$AA=\left(\frac{Rcontrol-Rsample}{Rcontrol}\right)\times 100$$

### Strengths and Limitations


Strengths:
Highly reproducible [112]Can screen both lipophilic and hydrophilic samples [114]
Limitations:
Time-consumingSometimes, the results from total bioactive components (phenolic, flavonoids) assay do not correlate with the results of this assayBad correlation with numerous assays, namely FRAP, CUPRAC, ABTS, and DPPHDifficulty in interpreting results in the presence of bad correlationsLow reproducibility [113]Sensitive to oxygen, pH, temperature, and solvent effects [111,112]



## 18. Conclusions

The science community is always avid for new technologies, new solutions, new applications, new methodologies, new products, and so on. This keen interest is for better advancement to provide more reliable and efficient results. After conducting a thorough literature search for the compilation of this present review, we noticed that the existing in vitro antioxidant assays possessed numerous controversies affecting their reliability. Presently, a universal and optimized protocol for the determination of antioxidant capacities is lacking. We do not have a standard assay that can give us an overall image of the antioxidant capacity that a test sample possesses. However, a combination of a few assays (minimum three) must be carried out to have a realistic assessment of the antioxidant capacity that a sample exhibit. This has been found to be time-consuming and expensive, since many reagents are needed. 

As discussed earlier in this review, different assays follow either the HAT or the SET mechanism or chelation of metal ions. Interestingly, a few antioxidant methods involve specific theories in addition to the type of mechanism they follow. For instance, the β-carotene assay is directly linked to the Polar Paradox Theory, since the assay is conducted in an emulsified medium and the potassium ferricyanide assay is related to the Crystal Field Theory (CFT) due to its octahedral structure surrounded by ligands (CN^−^). As expected, antioxidant assays hold a lot of chemistry. During the last few years, shreds of evidence have piled up against the methods used to measure antioxidant activity in biological samples. Several reviews have been published with considerably different opinions. It seems that there is no consensus of opinions among scientists, most probably because the area of antioxidants is such a complex topic. Since a standardized and thoroughly validated assay is lacking, it is difficult to make a comparison between results gathered by different research groups. It is also difficult to conduct suitable quality control for antioxidant products in food and nutraceutical industries [8]. In addition, existing published results remain questionable and inconclusive. Usually, after a preliminary screening (in vitro analysis) is performed, scientists tend to embark on in vivo testing and later, in clinical research. In that sense, if there is a serious shortfall in in vitro results, the following in vivo analysis together with advanced clinical research can be compromised, which represents a major hurdle. 

Based on the existing literature, the current antioxidant assays have been reported to possess several strengths such as simple procedure, rapid analysis time, screening of many samples in a timely fashion, cheap reagents, and simple instrumentation being used. These strengths are generally good but not strong enough to support the efficacy and reliability of these assays as they are seriously flawed. Overall, antioxidant methods are non-specific, non-sensitive, bear no resemblance to biological systems, have bad correlation with bioactive compounds, and reaction of other species to react with the oxidant leading to overestimation is possible, to cite a few. To be able to develop a universal and reliable antioxidant assay, these limitations can be addressed by answering the following questions: (1) What are the real protective properties of antioxidants? (2) What type of species in these antioxidant assays can provide protection or which substrates are being oxidized and what products will inhibit the oxidation? (3) Is there any possible effect caused by other chemical species present in the system under investigation? (4) What can be done to improve the similarity of these antioxidant assays to biological systems, i.e., how to reduce the gap between in vitro methods and in vivo experiments? (5) Under which conditions we should work to improve the sensitivity and specificity? (6) How can underestimation and overestimation of antioxidants be prevented? (7) How can poor correlation between bioactive components (phenolic, flavonoid) and antioxidants be solved? Huang, Ou, and Prior [8] have mentioned that if we claim antioxidant activity of tested samples solely based on assays such as DPPH, ABTS, FRAP, and CUPRAC, it would be unscientific, exaggerated, and out of context, since they bear no similarity to biological systems. 

**Figure 19:**
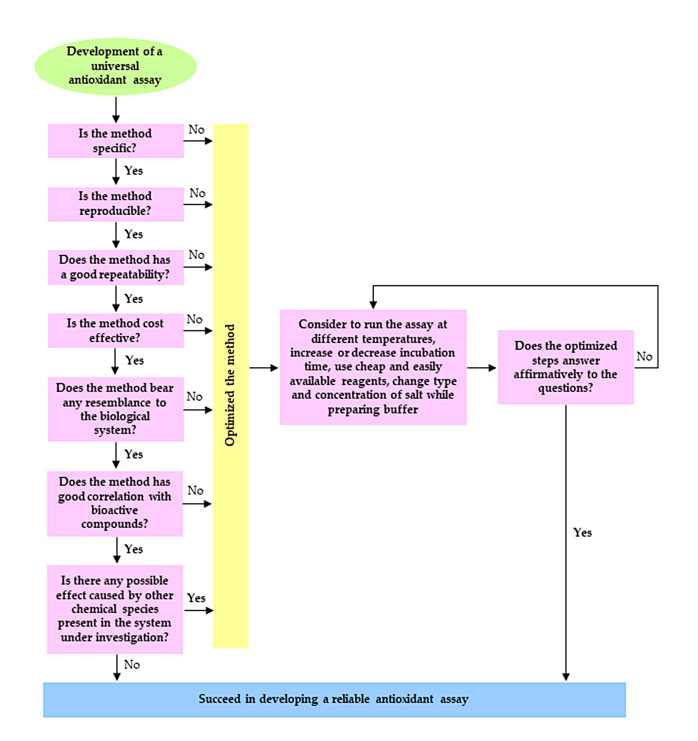
Flowchart with proposed steps to follow in order to develop a universal antioxidant assay.

In conclusion, it is of paramount importance to develop a proper and universal antioxidant method emphasizing the fundamental chemistry rather than concentrating on the development of an easy, simple, economical, and rapid assay with the aim to better elucidate radical quenching, reducing capacity or metal chelation ability of different groups of compounds present in biological samples. Figure 19 illustrates a flowchart showing important steps in the development of a universal antioxidant assay in a sequential order. Finally, we must learn how to exploit this information for a more effective application in the clinical context. 

## Figures and Tables

**Table 1:** Different techniques used to measure antioxidant activity (Source: [20]).

Antioxidant Assay	Principle of Method	End Product Determination
**Spectrometry**		
DPPH	Antioxidant reaction with an organic radical	Colorimetry
ABTS	Antioxidant reaction with an organic radical	Colorimetry
FRAP	Antioxidant reaction with a Fe (III) complex	Colorimetry
PFRAP	Potassium ferricyanide reduction by antioxidants and subsequent reaction of potassium ferrocyanide with Fe^3+^	Colorimetry
CUPRAC	Cu (II) reduction to Cu (I) by antioxidants	Colorimetry
ORAC	Antioxidant reaction with peroxyl radicals, induced by AAPH	Loss of fluorescence of fluorescein
HORAC	Antioxidant capacity to quench OH radicals generated by a Co (II) based Fenton-like system	Loss of fluorescence of fluorescein
TRAP	Antioxidant capacity to scavenge luminol-derived radicals, generated from AAPH decomposition	Photo chemiluminescence quenching
Fluorimetry	Emission of light by a substance that has absorbed light or other electromagnetic radiation of a different wavelength	Recording of fluorescence excitation/emission spectra
**Electrochemical Techniques**		
Cyclic voltammetry	The potential of a working electrode is linearly varied from an initial value to a final value and back, and the respective current intensity is recorded	Measurement of the intensity of the cathodic/ anodic peak
Amperometry	The potential of the working electrode is set at a fixed value with respect to a reference electrode	Measurement of the intensity of the current generated by the oxidation/reduction of an electroactive analyte
Biamperometry	The reaction of the analyte (antioxidant) with the oxidized form of a reversible indicating redox couple	Measurement of the current flowing between two identical working electrodes at a small potential difference and immersed in a solution containing the analysed sample and reversible redox couple
**Chromatography**		
GC	Separation of the compounds in a mixture is based on the repartition between a liquid stationary phase and a gas mobile phase	Flame ionization or thermal conductivity detection
HPLC	Separation of compounds in a mixture is based on the repartition between a solid stationary phase and a liquid mobile phase with different polarities, at high flow rate and pressure of the mobile phase	UV–vis (e.g., diode array) detection, fluorescence, mass spectrometry or electrochemical detection
TLC	Separation of compounds is based on the repartition between a solid stationary phase (silica gel) and a liquid mobile phase (mixture of acetate, formic acid and water)	Photographed under visible light

DPPH—2,2-diphenyl-1-picrylhydrazyl; ABTS—2,2-azino-bis(3-ethylbenzothiazoline-6-sulfonic acid); FRAP—Ferric reducing antioxidant power; PFRAP—Potassium ferricyanide antioxidant power; CUPRAC—Cupric reducing antioxidant capacity; ORAC—Oxygen radical absorbance capacity; HORAC—Hydroxyl radical antioxidant capacity; TRAP—Total radical trapping antioxidant parameter; GC—Gas chromatography; HPLC—High performance liquid chromatography; UV–vis—Ultraviolet–visible; TLC—Thin layer chromatography; AAPH—2,2′-azobis-2-amidino-propane.
